# Mechanistic Insight into H_2_S‐Induced Fluorescence Quenching in a Robust Metal–Organic Framework

**DOI:** 10.1002/smll.74030

**Published:** 2026-06-02

**Authors:** Valeria B. López‐Cervantes, Juan L. Obeso, J. Gabriel Flores, Ricardo A. Peralta, José Antonio de los Reyes, Jiangnan Li, Sihai Yang, Elodie Strupiechonski, Andrés De Luna Bugallo, Aída Gutiérrez‐Alejandre, Yoarhy A. Amador‐Sánchez, Diego Solis‐Ibarra, Ilich A. Ibarra

**Affiliations:** ^1^ Laboratorio de Fisicoquímica y Reactividad De Superficies (LaFReS) Instituto De Investigaciones en Materiales Universidad Nacional Autónoma De México Ciudad de México México; ^2^ College of Chemistry and Molecular Engineering Beijing National Laboratory For Molecular Sciences Peking University Beijing China; ^3^ Departamento de Ingeniería de Procesos e Hidráulica División de Ciencias Básicas e Ingeniería Universidad Autónoma Metropolitana‐Iztapalapa Ciudad de México México; ^4^ Área de Química Aplicada, Departamento De Ciencias Básicas Universidad Autónoma Metropolitana‐Azcapotzalco Ciudad de México México; ^5^ Departamento de Química División De Ciencias Básicas e Ingeniería. Universidad Autónoma Metropolitana‐Iztapalapa Ciudad de México México; ^6^ Centro de Ingeniería y Desarrollo Industrial Querétaro Mexico; ^7^ Centro de Física Aplicada y Tecnología Avanzada Universidad Nacional Autónoma De México Querétaro México; ^8^ UNICAT, Departamento De Ingeniería Química Facultad De Química Universidad Nacional Autónoma De México Ciudad de México México

**Keywords:** adsorption, fluorescent sensing, H_2_S, MOF, structure

## Abstract

The development of chemically robust sorbents capable of integrating capture and optical sensing of toxic gas remains a major challenge. Herein, the ultramicroporous Zn(II)‐based metal‐organic framework, MFM‐520, has been investigated for the adsorption and luminescent detection of H_2_S. Breakthrough measurements at 298 K and 1 bar show a reversible H_2_S uptake of 3.91 mmol g^−1^, while powder X‐ray diffraction confirms full retention of crystallinity after adsorption. Solid‐state photoluminescence experiments reveal a pronounced, selective fluorescence turn‐off response to H_2_S in the gas phase, with a limit of detection of 6.13 ppm. Time‐resolved spectroscopy shows a decrease in the excited‐state lifetime upon H_2_S adsorption, indicating enhanced non‐radiative decay pathways. In situ DRIFTS measurements indicate that H_2_S interacts with MFM‐520 through supramolecular interactions. The combined adsorption, structural, and spectroscopic analyses establish that fluorescence quenching arises from reversible, confinement‐amplified modulation of ligand‐centered excited‐state dynamics. These results highlight how coordinatively saturated Zn(II) nodes within ultramicroporous environments can couple chemical stability with selective luminescent gas sensing.

## Graphical abstract

An ultramicroporous Zn‐based metal‐organic framework, MFM‐520, enables reversible hydrogen sulfide adsorption and selective fluorescence sensing. Confinement of H_2_S within the pores perturbs ligand‐centered excited states through weak host‐guest interactions, enhancing non‐radiative decay pathways. This guest‐induced modulation of excited‐state dynamics produces a pronounced fluorescence turn‐off response toward H_2_S.

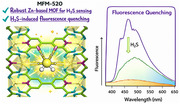

## 1 Introduction

Hydrogen sulfide (H_2_S) is a highly toxic gas classified as a primary air pollutant, commonly present in natural gas and biogas streams and it is also emitted from various industrial processes, including oil refineries and wastewater treatment plants [1]. H_2_S is a colorless, hazardous, and flammable gas that is particularly corrosive, which can lead to severe material degradation and, upon atmospheric oxidation, contributes to the formation of secondary pollutants associated with acid rain [2]. Even at relatively low concentrations (e.g., ppm levels), H_2_S is extremely harmful to human health, causing severe nervous system failure and respiratory paralysis [3]. Therefore, the efficient capture and reliable monitoring of H_2_S are of critical importance for environmental protection and human health [4, 5]. In this regard, sorbent materials that combine high adsorption affinity with adsorption‐driven sensing mechanisms are urgently needed.

**FIGURE 1:**
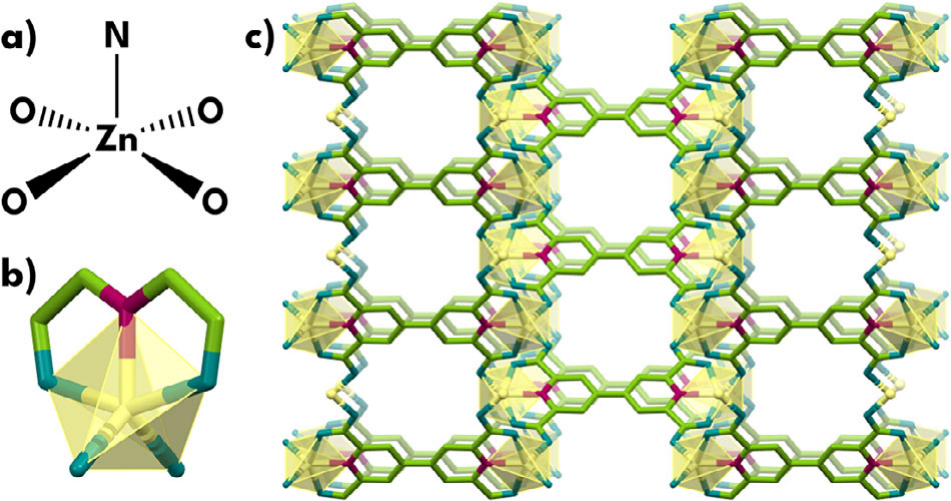
Structural representation of MFM‐520: (a) square‐pyramidal pentacoordinated Zn(II) center; (b) local view of the [ZnO_4_N] node; and (c) extended framework viewed along the b axis. Color code: Zn, yellow polyhedra; C, green; O, cyan; N, magenta.

Current technologies for capturing H_2_S include ionic liquids, alkanol amines (reactive and non‐reactive absorption), cryogenic sequential distillation [6], and physisorption in zeolites, activated carbons, and metal oxides [7]. However, such approaches face challenges, including pipeline corrosion, very low H_2_S capture, large amounts of wastewater, high re‐use costs, low recovery, and, for porous solid materials, significant loss of porosity due to required high re‐activation temperatures [8, 9]. In parallel, the most widely used H_2_S detection technologies are based on electrochemical sensors employing metal oxides [10], which are often susceptible to poisoning by interfering species present in real environments, leading to poor selectivity and loss of sensitivity over time. Metal‐organic frameworks (MOFs) have attracted significant attention for their potential to integrate efficient H_2_S capture and detection within a single material platform [11]. Due to their high porosity, chemical stability, functionalization, low‐cost reactivation, and well‐defined distribution of metal centers, MOF materials exhibit outstanding H_2_S uptakes [12]. For example, MIL‐53(Al, Cr, Fe), MIL‐47(V), MIL‐100(Cr), and MIL‐101(Cr) have been applied to H_2_S removal [13]. Beyond adsorption, MOFs are promising materials for advanced H_2_S detection. For instance, H_2_S sensing has been demonstrated by incorporating MOF materials into multifunctional electronic textiles [14]. Also, luminescent MOFs offer an attractive sensing strategy, as interactions between guest molecules and the framework can induce measurable changes in fluorescence intensity or spectral profile [15]. In this context, recent studies have demonstrated H_2_S detection using chemically stable MOFs through diverse strategies, including reactive “turn‐on” sensing based on chemical transformation (e.g., azide‐to‐amine conversion) [16], luminescent Zr‐based frameworks for aqueous detection of H_2_S or S^2−^ [17], and MOF‐based mixed‐matrix membranes (MMMs) that improve processability and sensing performance [18]. In addition, post‐synthetically modified MOFs have enabled sensing in biological environments [19], while lanthanide‐based systems offer multi‐responsive detection through modulation of emission pathways [20]. However, most reported systems rely on chemical reactivity, operate in solution, or lack a detailed mechanistic understanding of the fluorescence response under gas‐phase conditions. Accordingly, establishing a direct correlation between H_2_S adsorption and fluorescence response remains challenging because highly chemically stable MOF materials are required. For example, due to the d^10^ electronic configuration, Zn(II) ion exhibits a highly flexible coordination environment, which readily adapts to intermediate and distorted coordination geometries [21], such as pentacoordinated or distorted octahedral, that favors the formation of cooperative and structurally persistent bonding units in MOFs, due to greater saturation of the coordination sphere and a reduction in labile sites susceptible to substitution [22, 23]. In that context, MFM‐520 material is constructed from pentacoordinated Zn(II) centers that adopt a square pyramidal geometry, described as a [ZnO_4_N] node, in which each Zn(II) ion is coordinated to a pyridine nitrogen atom and four carboxylate oxygens from different ligands (Figure 1). This connectivity generates a highly stable tridentate synthon, in which the set of O and N donors cooperates to effectively saturate the metal coordination sphere, suppressing the lability typical of many Zn(II) nodes in porous frameworks. As reported in detailed structural studies of MFM‐520, the material exhibits meta‐rigid behavior, in which the [ZnO_4_N] node can undergo reversible geometric distortions without metal‐ligand bond breakage attributable to angular relaxation of the Zn–O/N environment [24]. This ability to accommodate local stresses while keeping the overall network intact is critical to its high stability. In this work, the ultramicroporous and chemically stable Zn(II)‐based MOF MFM‐520, [Zn_2_(L)] (H_4_L = 4,4´‐bipyridine‐2,2´,6,6´‐tetracarboxylic acid) has been selected to investigate H_2_S adsorption and detection. Owing to its structurally saturated [ZnO_4_N] nodes and bowtie‐shaped ultramicropores (dimensions of 6.6 × 4.0 Å^2^) [25, 26], MFM‐520 provides a confined environment in which weak host‐guest interactions can be amplified without compromising framework integrity. We hypothesized that, rather than inducing direct metal coordination or structural degradation, H_2_S would interact with the aromatic linker via supramolecular contacts, thereby modulating ligand‐centered excited‐state dynamics. By combining breakthrough experiments, in situ DRIFTS analysis, and steady‐state and time‐resolved photoluminescence spectroscopy, we have established a direct correlation between H_2_S adsorption and fluorescence response, and to elucidate the mechanism by which spatial confinement translates weak intermolecular interactions into measurable changes in emission behavior.

## 2 Results and Discussion

### 2.1 MFM‐520 Characterization

MFM‐520 was synthesized following a previously reported method [25]. Phase purity of MFM‐520 was corroborated using powder X‐ray diffraction (PXRD) and Fourier‐transform infrared (FTIR). The PXRD pattern of MFM‐520 shows well‐defined characteristic peaks, indicating high crystallinity [25].

### 2.2 H_2_S Adsorption Performance

**FIGURE 2:**
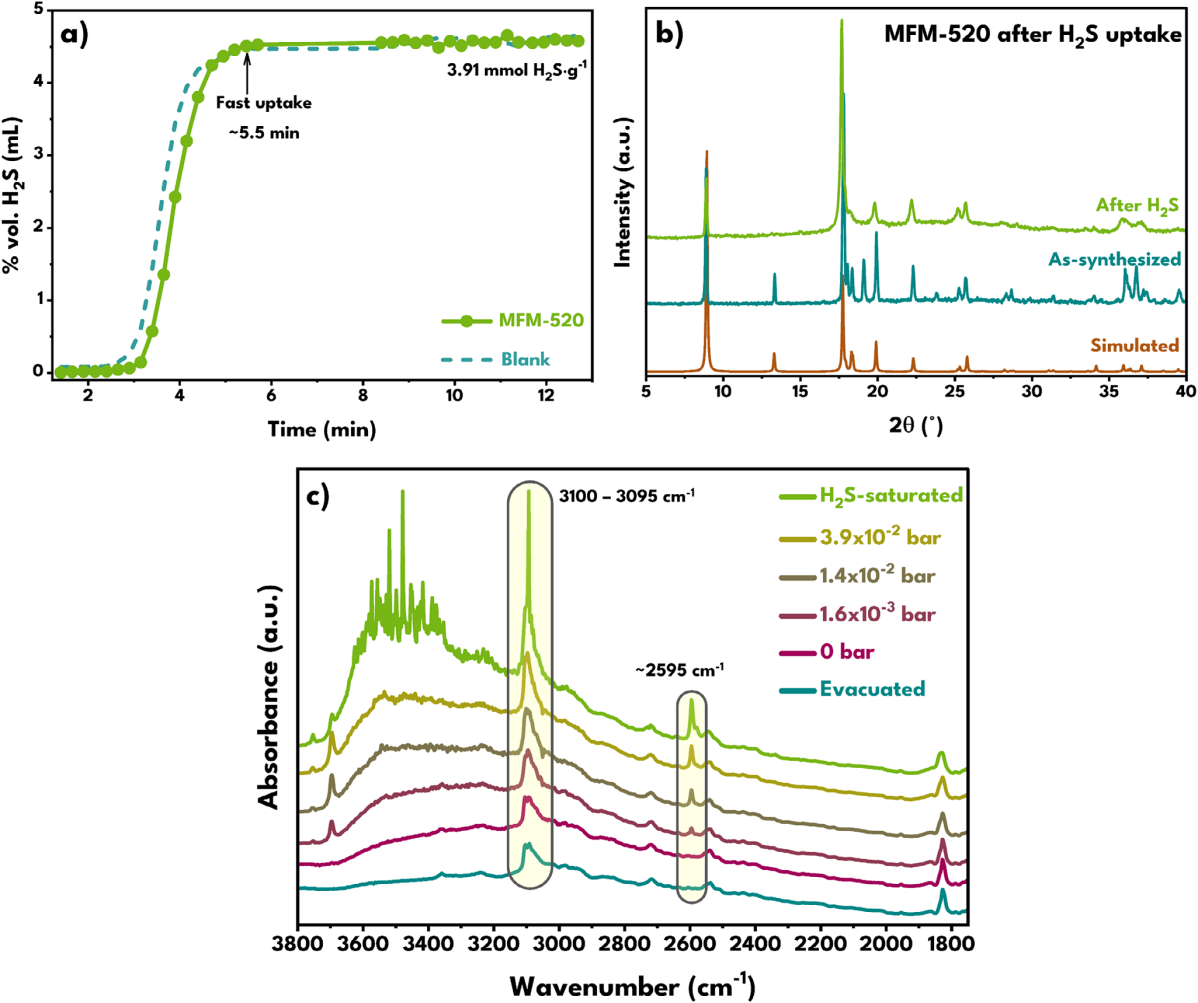
(a) H_2_S adsorption isotherm of MFM‐520 compared with a blank measurement. (b) PXRD patterns of simulated, as‐synthesised, and H_2_S‐exposed MFM‐520. (c) *In situ* DRIFTS spectra of activated MFM‐520 under different H_2_S pressures at 298 K, showing the appearance of the S─H stretching band (~2595 cm^−1^) together with perturbations in the aromatic C─H region, consistent with weak host‐guest interactions within the ultramicroporous framework.

The dynamic H_2_S adsorption performance of MFM‐520 was evaluated using breakthrough experiments. The measurements were carried out using an H_2_S concentration of 5 vol% in 95 vol% N_2_. Prior to adsorption, an acetone‐exchanged MFM‐520 was activated at 453 K for 2 h under a flow of dry N_2_. The breakthrough curves of H_2_S adsorption (Figure 2a) reveal a high dynamic uptake capacity of 3.91 mmol g^−1^. This highlights the strong affinity of MFM‐520 toward H_2_S under flow conditions. Interestingly, this uptake surpasses that of leading MOF materials, such as Mg‐CUK‐1 (1.41 mmol g^−1^) [27], MIL‐101(Cr) (0.4 mmol g^−1^) [28], and Zn‐MOF‐74 (1.6 mmol g^−1^) [28].

The full retention of structural integrity of MFM‐520 after the H_2_S breakthrough experiment was confirmed by PXRD (Figure 2b), demonstrating the excellent chemical robustness of MFM‐520. The resistance of MFM‐520 to H_2_S can be understood by applying Pearson's hard and soft acids and bases (HSAB) principle to the coordination environment of Zn(II) [29]. Zn(II) is a borderline acid, and in MFM‐520 it is stabilised by a combination of hard bases (carboxylate oxygen atoms) and slightly soft bases (pyridine nitrogen atoms), resulting in an electronically balanced node that is strongly anchored to the ligand [30]. In this context, H_2_S, which is a soft and neutral base under non‐dissociating conditions, lacks the thermodynamic strength necessary to compete with the cooperative O, O, N set that defines the tridentate synthon [30]. Furthermore, the effective saturation of the Zn(II) coordination sphere prevents the possibility of unsaturated sites for direct Zn‐S interaction, making adsorption dominated by intermolecular interactions within the pore [31]. Thus, the combination of a frontier‐metal acid and a mixed hard/frontier‐donor environment consistently explains why MFM‐520 can tolerate H_2_S without structural degradation. The activated MFM‐520 exhibits a BET surface area of 308.2 m^2^ g^−1^, in excellent agreement with the literature value reported for the same framework ([Zn_2_(L)]) (312.7 m^2^ g^−1^). After H_2_S adsorption/desorption, the BET surface area remains essentially unchanged (310.7 m^2^ g^−1^), confirming preservation of the intrinsic ultramicroporosity and structural integrity of the framework.

The interactions between H_2_S and the MFM‐520 framework are dominated by intermolecular interactions, which are favored by the strict spatial confinement within the ultramicroporous pores. MFM‐520 exhibits pores with dimensions of 6.6 × 4.0 Å^2^, comparable to the kinetic diameter of H_2_S (∼3.6 Å) [32], allowing efficient accommodation of the molecule within the channel without inducing significant structural stresses in the framework. Under these confinement conditions, the gas is sufficiently close to the aromatic fragments of the ligand to establish directional supramolecular interactions, such as C─H···S and induced dipole–dipole. The latter is consistent with in situ DRIFTS experiments. In situ DRIFT experiments were performed at 298 K and atmospheric pressure to investigate the interaction between H_2_S and the MFM‐520 framework. The IR spectra for MFM‐520 samples are displayed: before (activated sample) and after H_2_S adsorption (under an atmosphere of H_2_S at different pressures) are displayed in Figure 2c. The bands observed at 3100–3095 cm^−1^ are related to aromatic C─H stretching vibrations of the organic linker [24]. Upon exposure to H_2_S, a characteristic new band at 2595 cm^−1^ is observed, corresponding to the S─H stretching vibration of gaseous H_2_S. The gradual decrease of this band with decreasing H_2_S pressure indicates a reduction in free gas‐phase H_2_S within the cell. Interestingly, slight changes are also observed in the aromatic C─H stretching region. This indicates that the H_2_S molecule perturbs the electronic environment of the linker. Thus, these observations suggest that H_2_S interacts with aromatic moieties within MFM‐520, likely via hydrogen bonding between the free electron pair on the S atom and the H atom of the CH group (C─H···S) and via induced dipole–dipole interactions [33]. Such interactions are consistent with reversible adsorption behavior.

**FIGURE 3:**
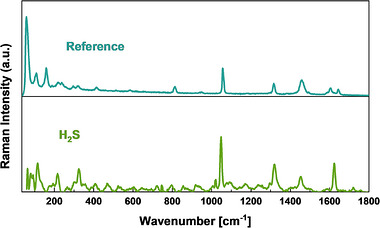
Raman spectra of MFM‐520 before and after H_2_S exposure, evidencing the absence of Zn─S bond formation and preservation of the framework structure.

As shown in the Raman spectra (Figure 3), the main ligand‐associated modes, particularly the aromatic ring breathing mode (∼1000 cm^−1^) and the C═C stretching region (1200–1600 cm^−1^) remain clearly observable after H_2_S exposure, indicating that the framework integrity is largely maintained.

H_2_S may exhibit affinity toward Zn^2+^ centers, and the possibility of Zn─S interactions must be considered. However, the Raman data provide important insight into the nature of this interaction. The most significant spectral changes occur in the low wavenumber region (<150 cm^−1^), which corresponds to collective lattice vibrations and framework modes in the terahertz (THz) regime, rather than localized bond‐stretching vibrations. These low‐frequency modes involve cooperative motions of the metal nodes and organic linkers and are highly sensitive to weak perturbations such as guest adsorption, pore filling, or changes in intermolecular interactions. The observed attenuation and broadening of these bands upon H_2_S exposure therefore indicate a modification of the global dynamical response of the framework, rather than the formation of specific, well‐defined Zn─S bonds. It is worth noting that no new Raman bands attributable to Zn─S stretching modes are observed upon H_2_S exposure. Such modes, if associated with strong chemisorption or bond formation, would be expected to appear as well‐defined features in the low‐to‐mid frequency region. Instead, the spectral response is characterized by an increased damping of collective modes, which can be attributed to weak interactions and dynamic disorder within the pores.

### 2.3 Photophysical Properties of MFM‐520

**FIGURE 4:**
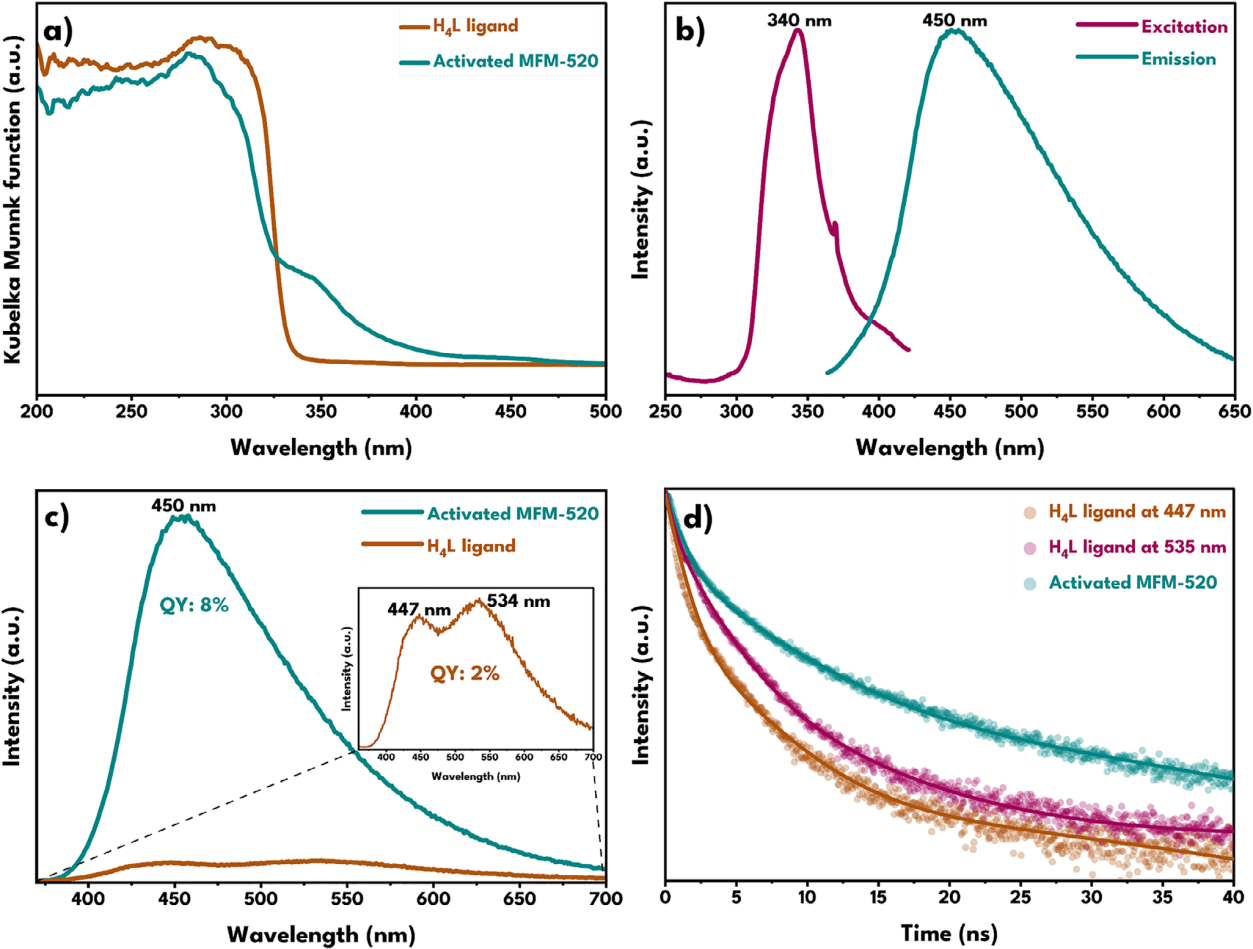
(a) UV–vis diffuse reflectance spectra of H_4_L and activated MFM‐520. (b) Excitation and PL spectra of activated MFM‐520. (c) Solid‐state PL spectra of H_4_L and activated MFM‐520 showing quantum yield enhancement upon coordination. (d) Time‐resolved photoluminescence decay profiles monitored at the corresponding emission maxima.

The UV–vis diffuse reflectance spectra of the H_4_L ligand and activated MFM‐520 were transformed using the Kubelka–Munk function, F(R), to obtain spectra proportional to the absorption coefficient (Figure 4a). The resulting profiles display an intense absorption onset in the near‐UV region with a sharp decrease around 360 nm, characteristic of allowed π→π* intraligand transitions within the aromatic framework. The absorption profile of the activated MOF displays a slight bathochromic shift and band broadening compared to the free ligand, indicating electronic modulation upon coordination to Zn(II). Importantly, no new absorption bands appear in the visible region, confirming that the optical response remains predominantly ligand‐centered and that Zn(II), owing to its d^10^ electronic configuration, does not introduce low‐lying metal‐centered transitions. These results indicate that the lowest excited states responsible for the emissive behavior originate from ligand‐based π→π* transitions.

The excitation and emission spectra of MFM‐520 in the solid state and after activation (Figure 4b) show behavior dominated by the organic ligand, where the excitation band has a well‐defined maximum around 340 nm, which can be attributed to π→π* intraligand electronic transitions. Meanwhile, the emission of the activated material is characterized by a broad band centered approximately at 450 nm. Based on these results, 340 nm was selected as the excitation wavelength for subsequent experiments.

The emission of the H_4_L acid ligand in the solid state, at λ_ex_ = 340 nm, shows a broad spectrum in the visible region with two maxima around 447 and 534 nm (Figure 4c), together with low quantum yield (QY ≈ 2%, Figure S14). In most aromatic organic systems, radiative deactivation occurs exclusively from the lower energy excited singlet state (S_1_), in accordance with Kasha's law [34]. However, the presence of two emission maxima in this case indicates a dual‐emission scenario, attributable to two distinct emitting singlet states (e.g., locally excited (LE) versus charge transfer (CT)) that do not fully interconvert before emission [35]. Thus, we can attribute the highest energy band at ∼447 nm to a localized π→π* intraligand transition, while the band with a maximum around 534 nm and greater spectral width may be due to a stabilized intraligand CT state. The presence of non‐bonding electron pairs in nitrogen atoms introduces excited states of the n→π* type that are close in energy to typical aromatic π→π* states, which favors vibronic coupling that opens channels for non‐radiative deactivation [36]. Additionally, the electro‐attractive behavior of –COOH groups can favor excited states of internal CT that also tend to deactivate fluorescence [37, 38]. All the above result in the poor quantum yield observed for the H_4_L ligand.

When the ligand coordinates with Zn(II), the photoluminescence of the ligand undergoes a significant reorganization in which the disappearance of the lower energy band at 534 nm is observed, suggesting that destabilization of the CT state occurs (Figure 4c). As a result, radiative deactivation occurs predominantly via intraligand π→π* transitions, giving rise to an intense, well‐defined emission band centered around 450 nm. On the other hand, the Zn(II) ion does not exhibit low‐energy d–d transitions that can serve as efficient channels for non‐radiative deactivation because of its d^10^ electronic configuration [39, 40]. Thus, the excited energy remains mainly localized in the ligand, favoring emission processes centered on the organic chromophore. In this way, Zn(II) essentially acts as a structural node that rigidifies and modulates the electronic environment of the ligand, resulting in an increase in QY to about 8% for the activated MFM‐520 (Figure S15) [41, 42]. Time‐resolved photoluminescence (TRPL) measurements further support the excited‐state reorganization induced by coordination. The free H_4_L ligand exhibits average lifetimes of 2.13 ns when monitored at 447 nm and 2.67 ns at 535 nm (λ_ex_ = 375 nm), confirming that both emissive bands arise from short‐lived singlet excited states (Figure 4d). The slightly longer lifetime associated with the 535 nm band is consistent with emission from a stabilized intraligand charge‐transfer (CT) state, which typically presents a more relaxed excited‐state geometry and enhanced non‐radiative decay channels. In contrast, activated MFM‐520 shows a significantly prolonged average lifetime of 5.57 ns when monitored at 450 nm, indicating a substantial suppression of non‐radiative deactivation pathways upon coordination to Zn(II). This lifetime extension is consistent with framework rigidification and destabilization of the CT state, as previously inferred from steady‐state emission data. Since Zn(II) possesses a d^10^ electronic configuration and does not introduce low‐energy metal‐centered states, the excited‐state energy remains predominantly localized on the ligand, while vibrational relaxation is reduced due to structural confinement within the framework. The increase in lifetime correlates directly with the enhancement of quantum yield observed for the activated MOF. Overall, the TRPL results demonstrate that coordination to Zn(II) enhances excited‐state stability and suppresses CT‐mediated non‐radiative decay, whereas guest interaction partially reactivates these relaxation pathways, directly linking structural modulation with photophysical response.

### 2.4 H_2_S Detection Measurements

**FIGURE 5:**
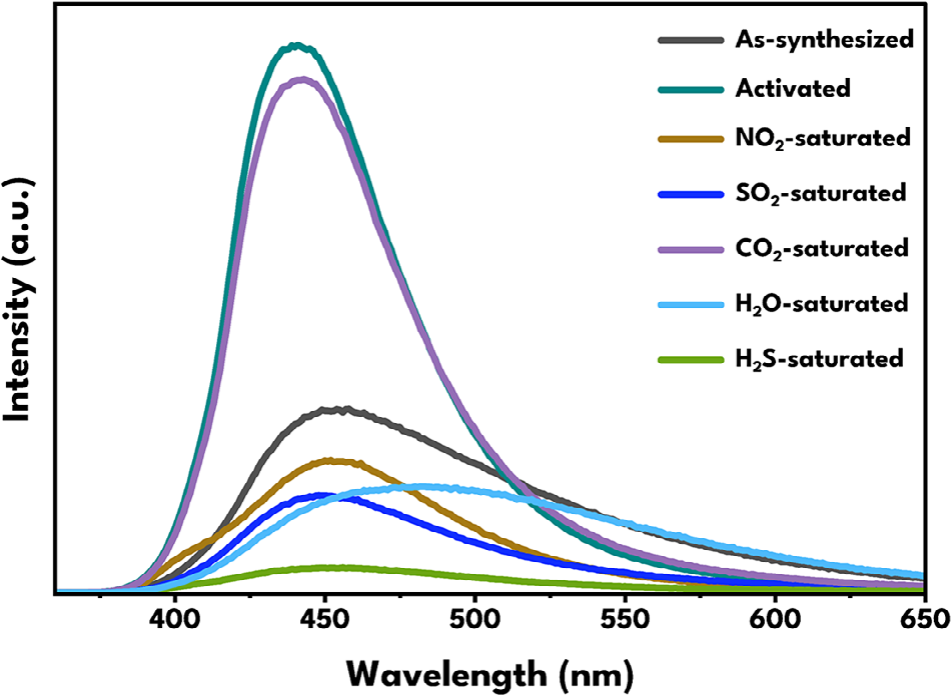
Solid‐state PL spectra of activated MFM‐520 toward different gaseous analytes (λ_ex_ = 340 nm).

The solid‐state photoluminescence response of activated MFM‐520 toward different gaseous analytes (Figure 5) reveals a gas‐dependent modulation of emission intensity centered at 447 nm (λ_ex_ = 340 nm). While most tested gases induce only minor variations in fluorescence intensity, exposure to H_2_S produces a distinctly stronger quenching effect. Interestingly, no significant shift in the emission maximum or alteration of the spectral profile is observed for any of the analytes, indicating that the emissive state retains its predominantly intraligand π→π* character and that no new emissive species is generated upon guest incorporation. The preservation of band shape suggests that the ground‐state electronic structure of the framework remains largely unaffected, in agreement with the UV–DRS results, and that the sensing response arises from modulation of excited‐state dynamics. The pronounced quenching observed for H_2_S therefore points to stronger guest–framework interactions relative to the other gases, likely involving specific interactions within the pore environment that enhance non‐radiative relaxation pathways. These findings demonstrate that MFM‐520 exhibits selective fluorescence quenching toward H_2_S over the other tested gaseous species, highlighting its potential as a luminescent sensor based on excited‐state perturbation mechanisms. While partial quenching is also observed for other gases, the significantly stronger response toward H_2_S indicates preferential host‐guest interactions; nevertheless, further studies under mixed‐gas conditions are required to fully assess selectivity under practical environments.

**FIGURE 6:**
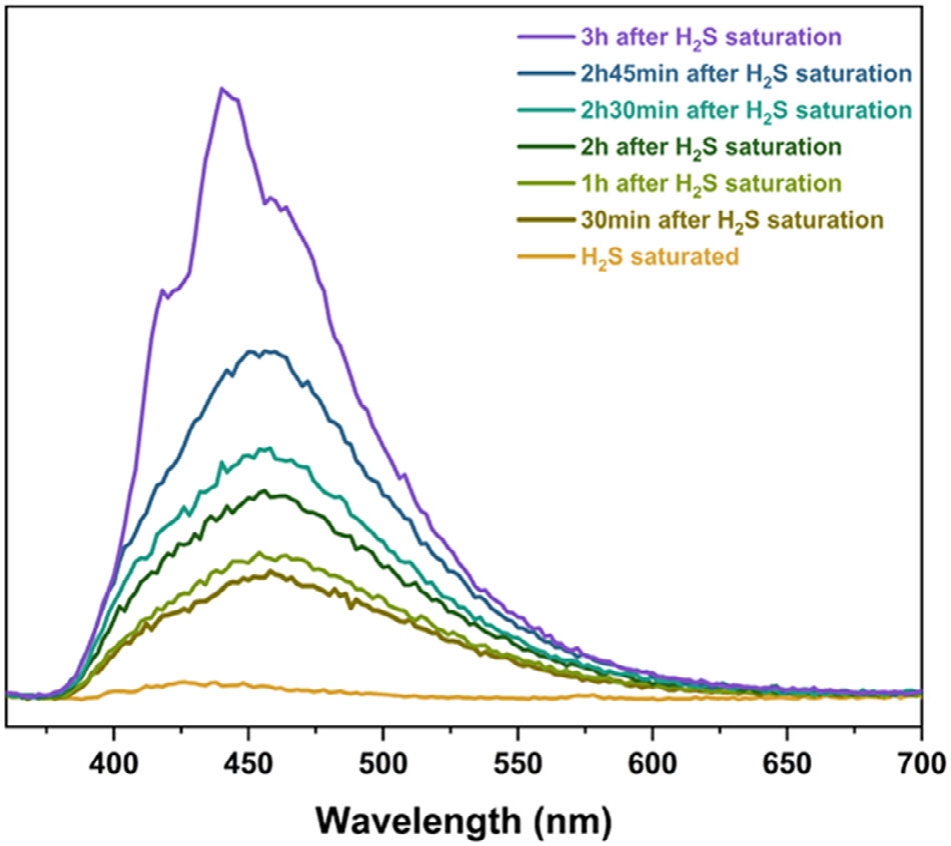
Time‐dependent recovery of the solid‐state photoluminescence of H_2_S‐saturated MFM‐520. PL spectra were recorded every 30 min over 3 h without re‐activation (λ_ex_ = 340 nm), showing gradual restoration of fluorescence intensity as H_2_S spontaneously desorbs from the framework pores. The emission maximum remains centered at ∼438–450 nm throughout the process, indicating preservation of the ligand‐centered emissive state.

H_2_S breakthrough experiments on MFM‐520 demonstrated complete reversibility of H_2_S adsorption without loss of framework crystallinity (*vide supra*). Based on this observation, we investigated the relationship between spontaneous H_2_S desorption and photoluminescence recovery as a function of time, without reactivating the sample. A H_2_S‐saturated MFM‐520 sample was monitored in situ by recording emission spectra every 30 min for 3 h (Figure 6).

A gradual increase in photoluminescence intensity was observed, indicating progressive desorption of H_2_S from the pores and concomitant recovery of the emissive state. The emission maximum remained essentially unchanged throughout this process, confirming that the recovery mechanism is governed by the removal of the quenching guest rather than by structural modification of the framework. The monitoring was extended until the emission intensity approached that of the as‐synthesized sample, which occurred after approximately one week (Figure S8), suggesting complete desorption of H_2_S from the pore environment. The complete recovery of the photoluminescence signal and the preservation of crystallinity suggest that the adsorption‐desorption process is fully reversible; however, further multi‐cycle studies are required to quantitatively evaluate the long‐term stability and reusability of the material.

Once the selectivity of MFM‐520 toward H_2_S over CO_2_, H_2_O, and SO_2_ was established, together with the reproducibility of the response under saturated conditions, we evaluated the photoluminescence response under lower H_2_S pressures. An activated sample was exposed to 0.1 bar of H_2_S, and the emission spectrum was recorded (Figure S9). The broad emission band remained centered at λ_max_ = 438 nm, consistent with ligand‐centered emission, while a decrease in intensity of approximately 70% was observed under these conditions. The reproducibility of the response was confirmed through five independent cycles of activation and re‐exposure to 0.1 bar of H_2_S, yielding a consistent average decrease of ∼41% in emission intensity. Although the experiments were performed under controlled pressure conditions and therefore cannot be directly extrapolated to trace‐level gas sensing, the consistent quenching behavior upon repeated exposure demonstrates the robustness of the photophysical response and highlights the potential of this chemically stable MOF for H_2_S detection.

To further evaluate the sensing capability of MFM‐520, the limit of detection (LOD) was determined using H_2_S dissolved in THF. The LOD was calculated as LOD = 3σ/m, where σ is the standard deviation of the emission intensity of activated MFM‐520 in THF and m is the slope of the linear fit obtained from concentration‐dependent quenching experiments. The LOD was determined in THF to enable controlled concentration‐dependent measurements; however, the fluorescence response observed under gas‐phase conditions confirms that the sensing mechanism is preserved upon H_2_S adsorption within the framework. A series of H_2_S solutions ranging from 0.5 to 100 mm was analysed (Figure S10), showing a clear linear correlation between emission intensity and H_2_S concentration (Figure S11). From this calibration curve, a limit of detection (LOD) of 0.18 mM was determined, corresponding to approximately 6.13 ppm. This result demonstrates that MFM‐520 enables reliable and quantifiable fluorescence quenching in the presence of H_2_S. Notably, this detection limit falls within the concentration range relevant for industrial safety and environmental monitoring; however, further improvements in sensitivity would be required to extend detection toward lower, trace‐level concentrations.

Some Zn‐based luminescent MOFs have been reported for H_2_S detection, including a methyl viologen‐templated Zn‐MOF capable of sensing H_2_S in natural gas with a detection limit of 28 nmol mL^−^^1^ (∼0.95 ppm) [43]. In contrast, the present study combines adsorption analysis, structural characterization, and time‐resolved spectroscopy to establish a direct correlation between H_2_S uptake and excited‐state dynamics, providing additional mechanistic insight into how weak host–guest interactions within ultramicroporous environments modulate ligand‐centered emission. A comparative summary of representative MOF‐based H_2_S sensors, including sensing conditions and performance metrics is provided in Table S3.

### 2.5 Quenching Mechanism in MFM‐520

**FIGURE 7:**
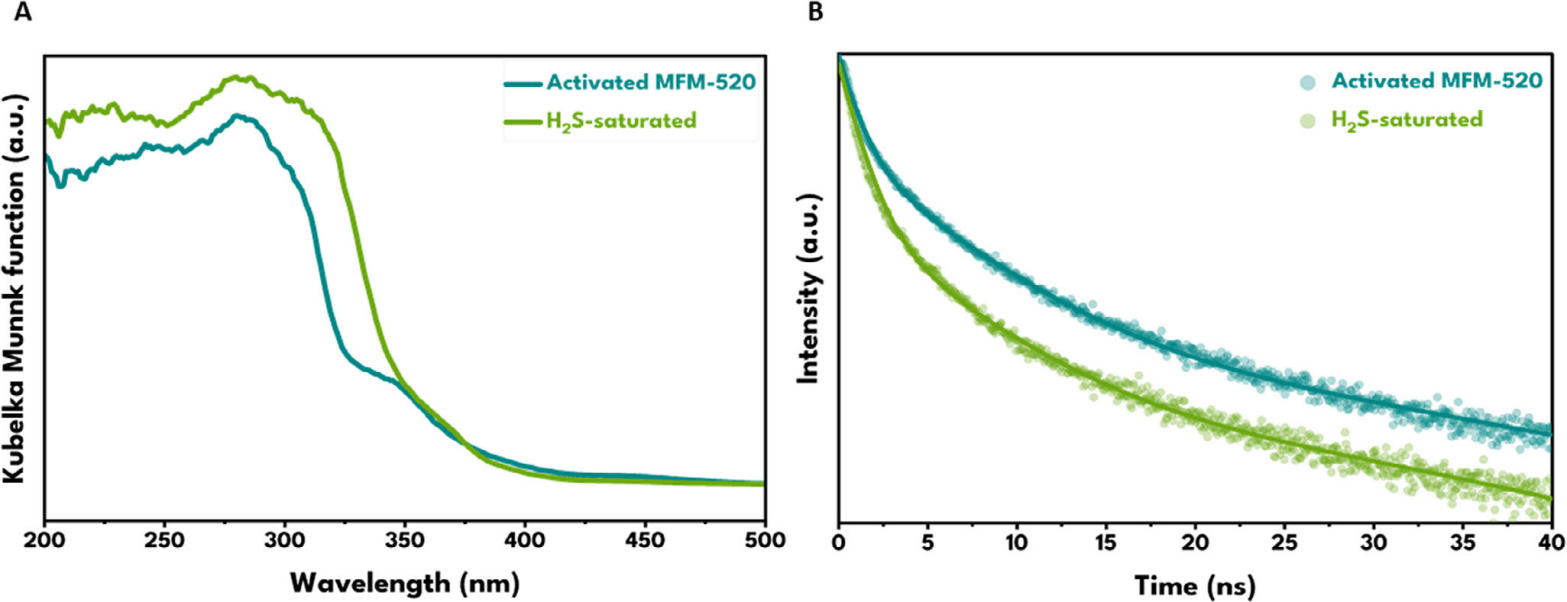
(A) UV–DRS spectra of activated MFM‐520 and H_2_S‐saturated MFM‐520, showing negligible changes in the ground‐state absorption profile after gas exposure. (B) Time‐resolved photoluminescence decay profiles (λ_em_ = 450 nm) of activated and H_2_S‐saturated MFM‐520, evidencing a decrease in the average excited‐state lifetime from 5.57 ns to 3.83 ns upon H_2_S adsorption.

Upon exposure to H_2_S, the quantum yield (QY) of MFM‐520 decreases markedly to 0.55%, evidencing highly efficient fluorescence quenching (Figure S16). The UV–DRS spectra (Figure 7A) show no significant change in the absorption profile after H_2_S saturation, indicating that the ground‐state electronic structure of the framework remains essentially unaltered. This observation rules out ground‐state complex formation or framework degradation as the origin of the sensing response and confirms that the quenching process is primarily governed by excited‐state perturbation. Since the emission of MFM‐520 is ligand‐centered in nature, the quenching behavior can be rationalized in terms of supramolecular interactions between adsorbed H_2_S molecules and the aromatic fragments of the organic linker. Within the confined ultramicroporous environment, weak host–guest interactions, including C─H···S contacts and induced dipole–dipole interactions, become spatially amplified. These interactions subtly perturb the electronic distribution of the chromophore, facilitating additional non‐radiative decay pathways. As a consequence, a significant fraction of the excited states undergo enhanced non‐radiative deactivation, resulting in substantial attenuation of emission intensity.

Time‐resolved photoluminescence measurements further clarify the quenching mechanism (Figure 7B). Upon H_2_S saturation, the average excited‐state lifetime decreases from 5.57 to 3.83 ns (monitored at 450 nm), directly confirming an increase in the non‐radiative decay rate (k_nr_). The concurrent decrease in both quantum yield and lifetime demonstrates that the quenching process contains a pronounced dynamic component rather than being purely static in origin.

Interestingly, the absence of spectral shifts in steady‐state emission, together with the retention of crystallinity and unchanged absorption features, indicates that the emissive state preserves its ligand‐centered character, and that no new emissive species is generated. In contrast to systems where preferential adsorption sites have been resolved crystallographically by in situ synchrotron PXRD [44], particularly in frameworks containing open metal sites, the interaction of H_2_S with MFM‐520 was demonstrated as relatively weak supramolecular interactions within the pore environment (Figure S13), as we previously demonstrated by in situ synchrotron X‐ray diffraction when SO_2_ is adsorbed within MFM‐520, finding the same preferential adsorption sites for H_2_S [24]. The absence of new metal‐sulfur vibrational modes, together with the reversible adsorption behavior and preserved framework structure, supports a physisorption‐dominated mechanism rather than chemisorption at the metal center.

Collectively, in situ DRIFTS experiments and steady‐state, time‐resolved, and diffuse reflectance results demonstrated that fluorescence quenching in MFM‐520 arises from reversible, guest‐induced modulation of excited‐state dynamics under spatial confinement. The ultramicroporous framework functions as a supramolecular amplifier, translating weak and reversible host–guest interactions into a measurable enhancement of non‐radiative relaxation pathways without altering the fundamental electronic structure of the material.

## 3 Conclusions

Finally, the Zn(II)‐based MOF, MFM‐520, has been demonstrated to combine efficient H_2_S adsorption with selective luminescent sensing within a single chemically robust platform. The material exhibits a high dynamic H_2_S uptake capacity (3.91 mmol g^−1^) under dynamic conditions while fully preserving its crystallinity, highlighting the structural stability of the pentacoordinated [ZnO_4_N] nodes toward this corrosive gas. In situ DRIFTS experiments indicate that H_2_S interacts with the framework through relatively weak supramolecular interactions rather than direct Zn‐S coordination, consistent with the saturated coordination environment and HSAB considerations. The strict spatial confinement within the 6.6 × 4.0 Å^2^ ultramicropores promotes guest–framework proximity, enabling efficient perturbation of the ligand‐centered excited state. Photophysical investigations reveal that coordination of the H_4_L ligand to Zn(II) enhances fluorescence efficiency by suppressing charge‐transfer‐mediated non‐radiative decay pathways, leading to increased quantum yield and prolonged excited‐state lifetime. Upon H_2_S adsorption, a pronounced and selective fluorescence quenching is observed without spectral distortion, confirming preservation of the ligand‐centered emissive state. Time‐resolved measurements demonstrate lifetime shortening upon guest incorporation, evidencing that the sensing response arises from guest‐induced modulation of excited‐state dynamics through enhancement of non‐radiative decay rates. The fluorescence response is fully reversible, recovering spontaneously upon H_2_S desorption without framework degradation, thereby establishing a direct correlation between adsorption and photophysical behavior. Additionally, a remarkable limit of detection of 0.18 mM (≈ 6.13 ppm) was achieved in solvent dispersion studies, further supporting the sensing capability of this material. Overall, this study demonstrates that a structurally saturated Zn(II) node combined with ultramicropore confinement enables the integration of chemical robustness for H_2_S adsorption and a excited‐state‐responsive luminescence within a single MOF platform. These findings provide valuable design principles for developing stable luminescent MOFs that couple gas capture and optical sensing by controlled modulation of excited‐state dynamics.

## 4 Experimental Section

### 4.1 Materials

All reagents and solvents were purchased from commercial suppliers and used without further purification. All water was deionized.

### 4.2 Synthesis

MFM‐520 was synthesized according to a previously reported solvothermal procedure [25]. In a typical preparation, Zn(II) salt and 4,4′‐bipyridine‐2,6,2′,6′‐tetracarboxylic acid (H_4_L) were dissolved in a mixed DMF/H_2_O solvent system and heated under autogenous pressure in a sealed vessel at elevated temperature for several hours. After slow cooling to room temperature, colorless crystalline solids were isolated by filtration, washed with fresh solvent, and dried under ambient conditions. Prior to gas adsorption and photoluminescence measurements, the as‐synthesized material was solvent‐exchanged with methanol and subsequently activated under dynamic vacuum at elevated temperature to afford the desolvated framework.

### 4.3 Analytical Instruments

A detailed description of the analytical instruments employed in this work is provided in the Supporting Information.

## Funding

I.A.I. thanks PAPIIT UNAM (IN201123), México, for financial support.

## Conflicts of Interest

The authors declare no conflicts of interest.

## Supporting information




**Supporting File**: smll74030‐sup‐0001‐SuppMat.docx.

## Data Availability

The data that support the findings of this study are available from the corresponding author upon reasonable request.
