# Supplementary material for: Mechanistic Insight into H2S‐Induced Fluorescence Quenching in a Robust Metal–Organic Framework
Source: Small. 2026 Jun 2;22(39):e74030. doi: 10.1002/smll.74030 (PMC13360734; doi:10.1002/smll.74030)
Supplement: Supplementary file 1 — Supporting File: smll74030‐sup‐0001‐SuppMat.docx. [file SMLL-22-e74030-s001.docx]

Supporting Information

Mechanistic Insight into H_2_S-Induced Fluorescence Quenching in a Robust Metal-Organic Framework

Valeria B. López-Cervantes,^+^ Juan L. Obeso,^+^ J. Gabriel Flores, Ricardo A. Peralta, José Antonio de los Reyes, Jiangnan Li, Sihai Yang, Elodie Strupiechonski, Andrés De Luna Bugallo, Aída Gutiérrez-Alejandre,* Yoarhy A. Amador-Sánchez,* Diego Solis-Ibarra* and Ilich A. Ibarra*

**S1. Analytical instruments**

**The powder X-ray diffraction (PXRD)** patterns were recorded on a Rigaku Diffractometer, Ultima IV, with Cu-Kα1 radiation (λ = 1.5406 Å) using a nickel filter. The patterns were recorded in the 2θ range of 2–50° with a step scan of 0.02° and a scan rate of 0.10 °/min.

**Solid-state ultraviolet-visible spectroscopy (UV-Vis)** was performed from 200-800 nm using a Shimadzu spectrophotometer UV-2600 equipped with an ISR-2600Plus integrating sphere and a BaSO_4_ blank.

**Fluorescence spectroscopy** was performed on an Edinburgh Instruments FS5 fluorimeter using a continuous wave 150 W ozone-free xenon arc lamp at room temperature, coupled with an SC-10 solid-state sample holder for solid-state measurements, and with an SC-05 standard cuvette holder for dispersed samples. For the emission experiments, the solid samples were slightly ground in an agate mortar to homogenize the microcrystals. They were later packed into the quartz sample holders and positioned in the instrument.

**Time-resolved photoluminescence (TRPL) spectra** were measured in an Edinburgh Instruments FS5 Spectrofluorometer using a 375 nm EPL laser, with an excitation bandwidth of 0.01 nm and an emission bandwidth of 5.00 nm at an emission wavelength of 450 nm.

**H_2_S Breakthrough experiments**

H_2_S experiments were made using an HP 5890 GC by continuous injections of the system exhaust; of each injection, we obtained a chromatogram. We integrate the H_2_S signal from the corresponding chromatogram to obtain its quantity. Knowing the H_2_S concentration from the feed, we can calculate the H_2_S concentration in each injection, as the saturation concentration is the original feed concentration. Dynamic breakthrough experiments were carried out in a homemade system (Scheme S1).


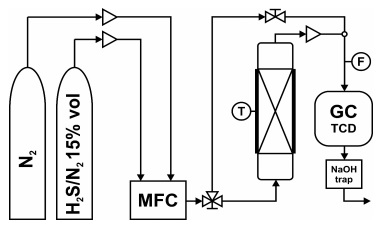


**Scheme S1.** Representation of breakthrough dynamic system for H_2_S uptake experiments.

Each sample was activated in situ at 80 °C for 2 hours with a constant flow of dry N_2_ (25 mL/min, ultrapure grade (99.98%) N_2_ gas (Praxair), and then slowly cooled to 25 °C. Then, the H_2_S concentration was adjusted with a mass flow controller fed with two lines: dry N_2_ and H_2_S/N_2_ 15 %vol. The gas concentration used for the H_2_S experiments was 10% of H_2_S with a flow of 25 ml/min. The breakthrough experiments were carried out at 25 °C, and the downstream flow was analyzed with a GC. The sample was reactivated at 25 °C for 15 minutes under a flow of dry N_2_ (25 mL/min).

The H_2_S adsorption capacity for each cycle was calculated using Eq. S1, where *‘V_H2S_’* represents the H_2_S volumetric capacity (cm^3^ g^-1^), *‘m’* the adsorbent mass (g), *‘F’* the input flow rate (cm^3^ min^-1^), *‘Cf’* and *‘Ct’* the influent and downstream H_2_S concentrations respectively (% vol), and *‘t’* the time (min).

$V_{H_{2}S}=\frac{F}{C_{f}m}\cdot\int_{0}^{t} \left( C_{f}-C_{t} \right)dt$ **Eq. S1**

As mentioned, the adsorption column has a porous glass bed; thus, a blank run before each experiment was measured to eliminate the column's adsorption contribution. Then the material corrected volumetric capacity ‘*V_H2S,corr_*’ was estimated using Eq. S2 for each cycle.

$V_{H_{2}S,corr}=V_{H_{2}S,blank}-V_{H_{2}S,sample}$ **Eq. S2**

The H_2_S adsorption capacity is often reported as *‘q_H2S_’* (mol g^-1^). This value was roughly estimated with the volumetric adsorption capacity ‘VH_2_S,corr’ (cm^3^ g^-1^) and the ideal gas law Eq. S3, Where ‘p’ is the system pressure (77.3 kPa), ‘T’ is the measurement temperature (298 K), and ‘R’ is the ideal gas constant (8314.4598 cm^3^ kPa K^-1^ mol^-1^).

$q_{H_{2}S}=\frac{V_{H_{2}S,corr \cdot p}}{R\cdot T}$ **Eq. S3**

**In situ DRIFTS of H_2_S**

DRIFT spectra of the MFM-520 were measured using an FTIR Nicolet 6700 spectrophotometer (DTGS detector) with a 4 cm^-1^ resolution equipped with a diffuse reflectance vacuum chamber with KBr windows. A pellet was made with the material, which was then placed in a closed system for degassing at 80 °C for 2 hours under a primary vacuum. Subsequently, the system was cooled and, upon reaching room temperature (material evaluated), was saturated with a mixture of H_2_S/N_2_ in a composition of 15% H_2_S up to a pressure of 29 bar. Then, under these conditions, DRIFT spectra were taken, and gradually, pressure was released by venting at the same time. Readings of the spectra were taken at each H_2_S pressure measured until reaching 0 torr with the help of primary vacuum.

**S2. Results and Discussions**

**MFM-520 characterization**

**Figure S1.** PXRD patterns of synthetized (purple) and reported (grey) MFM-520.

**H_2_S breakthrough experiment**

**Figure S2.** Dynamic breakthrough experiment for H_2_S adsorption on MFM-520 (298 K, 1 bar).

**Table S1.** H_2_S adsorption capacity for known MOF materials.

| **Material** | **H_2_S uptake in this work (mmol g^-1^)** | **H_2_S uptake reported (mmol g^-1^)** | **Ref.** |
| --- | --- | --- | --- |
| Mg-CUK-1 | 1.39 | 1.41 | ^[1]^ |
| MIL-53(Al)-TDC | 18.2 | 18.1 | ^[2]^ |

**H_2_S saturation experiments**

The system contains two principal parts:

1. The gas generator, in which Fe_2_S_3_ is added to a two-neck ball flask [1]. One of the ports is capped with a rubber stopper through which concentrated HCl is injected with a glass syringe [2], while the other port is connected to the saturation chamber.
2. The saturation chamber, made of a round flask [3], is connected to a vacuum line [4] and a pressure gauge [5].

To start the process, a sample of about 15 mg in a 1.5 mL glass vial was activated in a sand bath with N_2_ flow at 80 °C under vacuum for 2 h. The vial was placed in the saturation chamber, and the system was evacuated with a vacuum line. Next, H_2_S gas was generated by dripping concentrated HCl over Fe_2_S_3_, the sample was left continuously exposed to the gas for 24 to 72 hours.


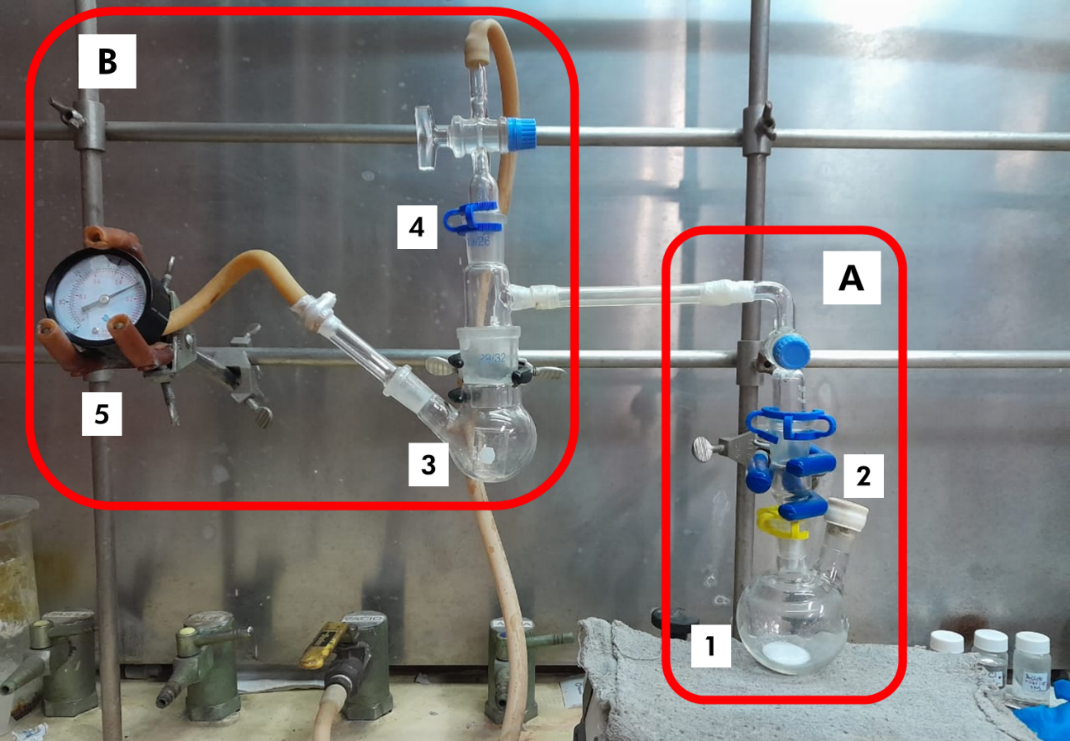


**Figure S3.** In situ H_2_S generation homemade system.

**Characterization after H_2_S sorption test**

**
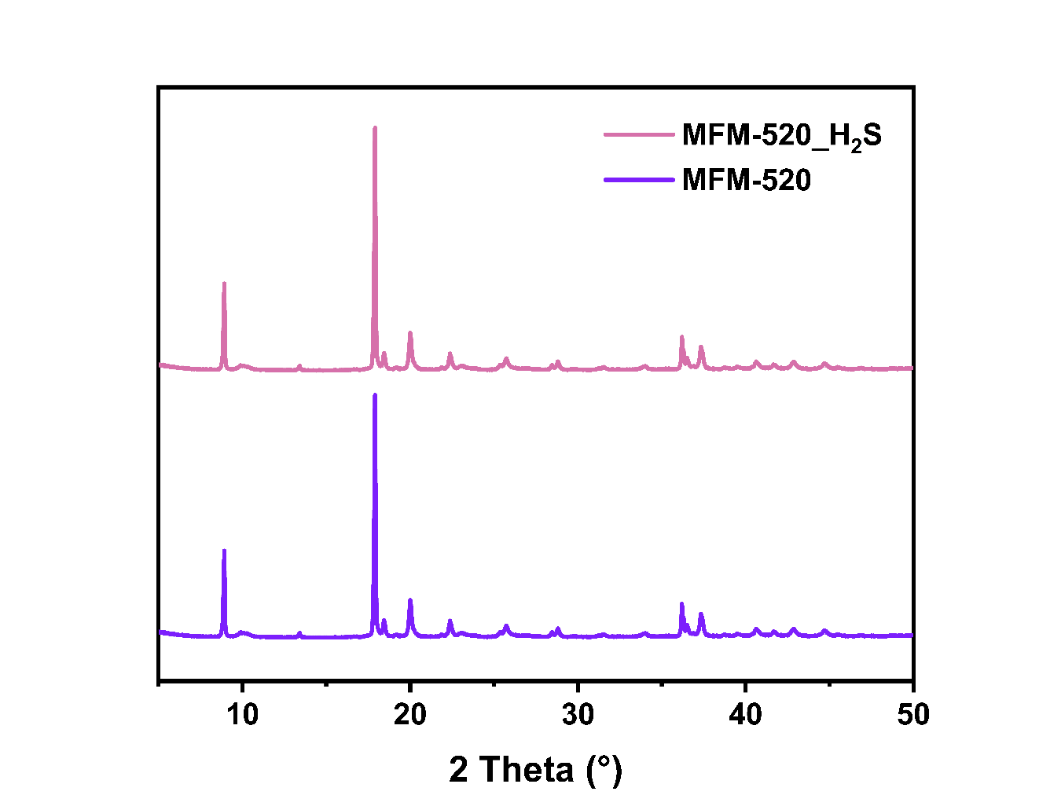
**

**Figure S4.** PXRD patterns of MFM-520 before (purple) and after the dynamic H_2_S adsorption experiments (pink).

**H_2_S sensing using MFM-520**


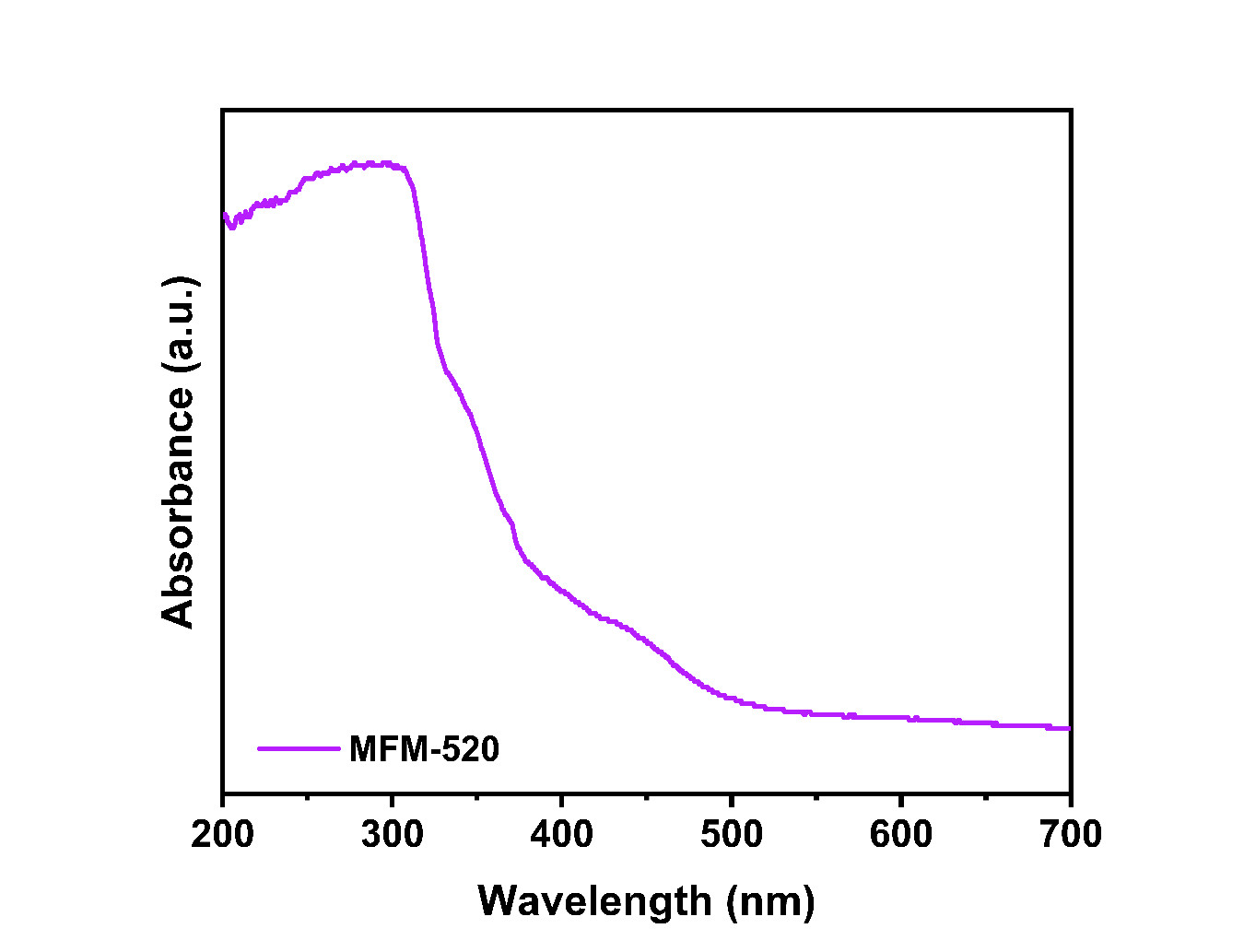


**Figure S5.** Solid-state UV-vis spectra of MFM-520.

**S3. Fluorescence spectroscopy**


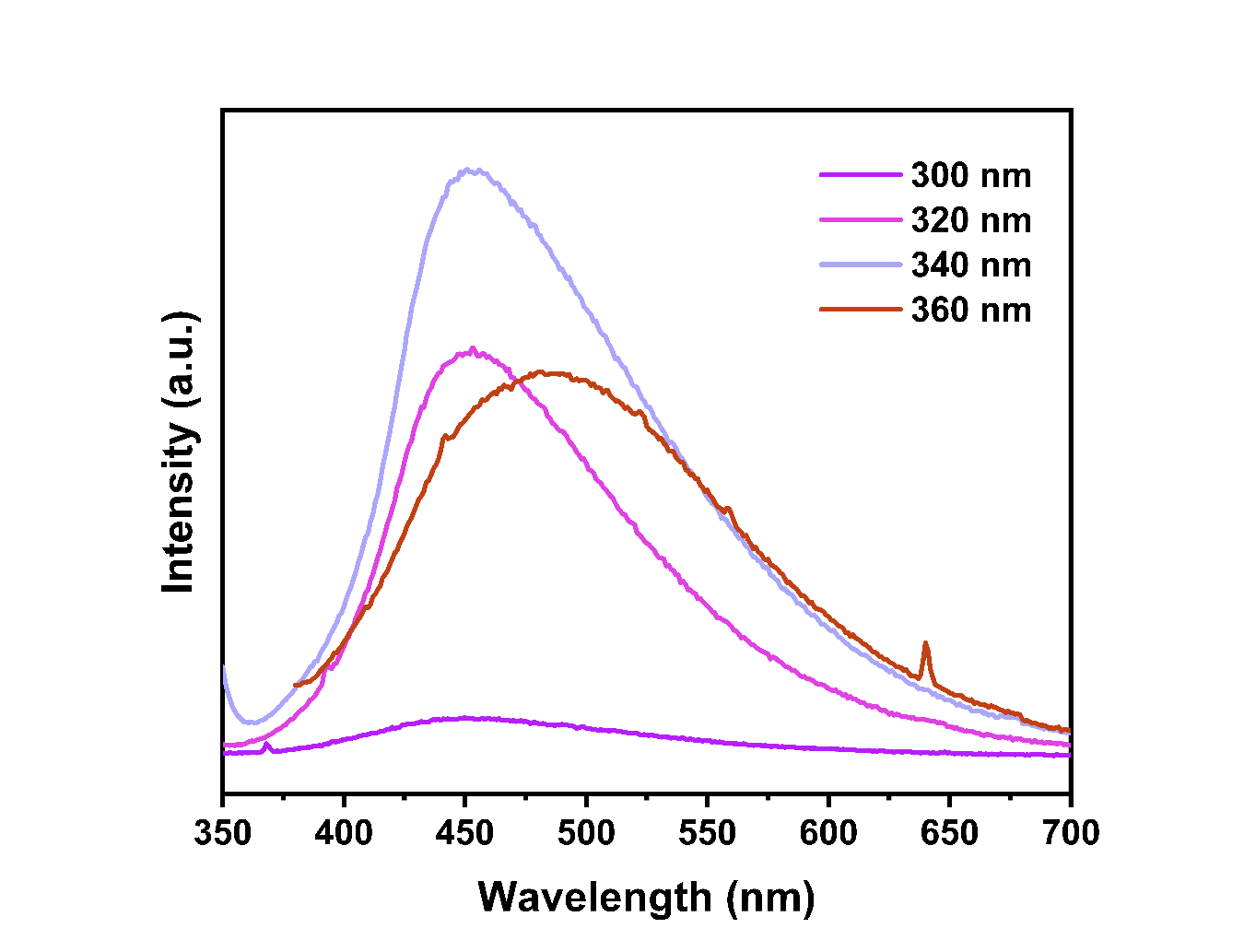


**Figure S6.** Solid-state PL spectra of MFM-520 recorded at different excitation wavelengths, showing preservation of the emission profile.


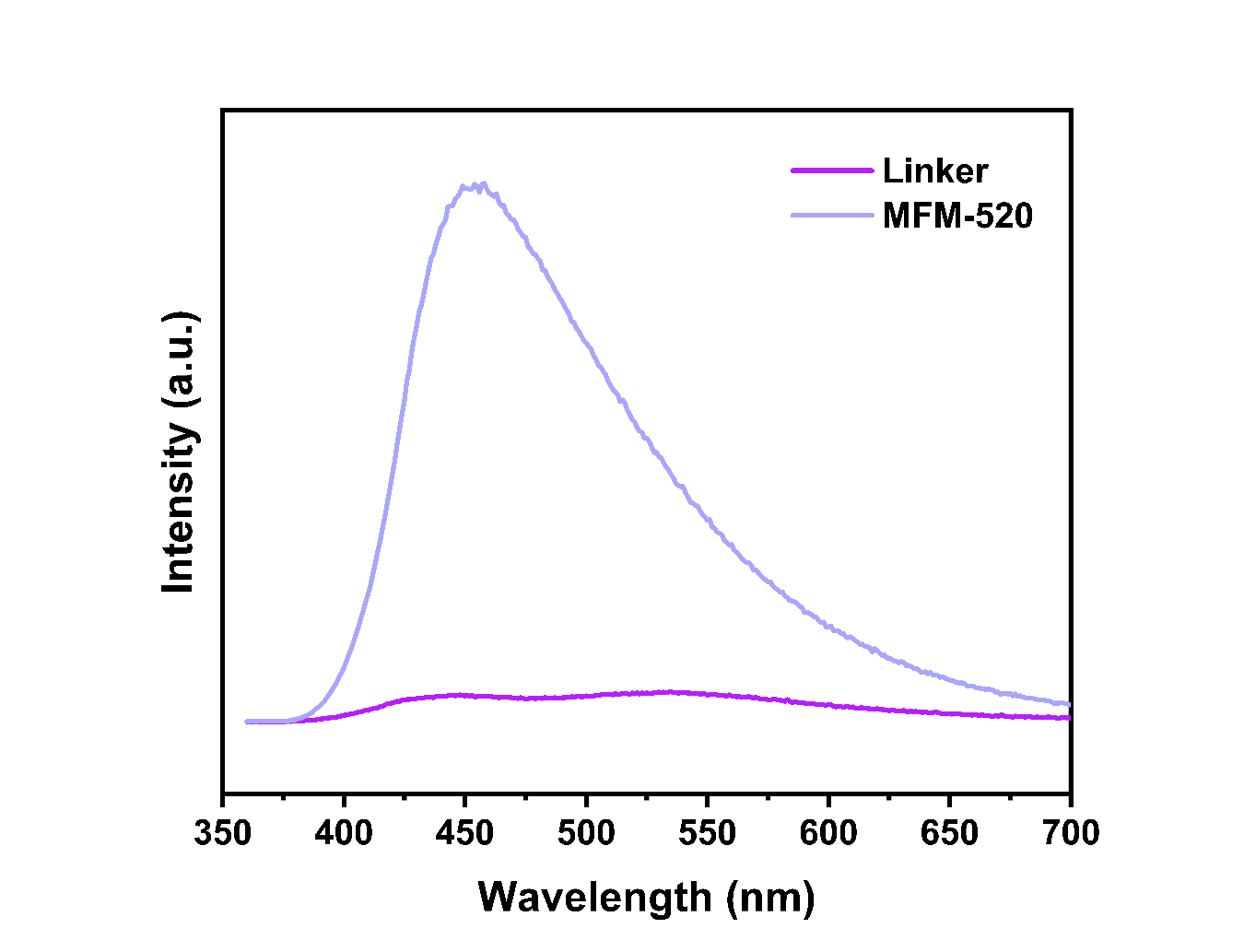


**Figure S7.** Solid-state PL spectra of the free linker and activated MFM-520.


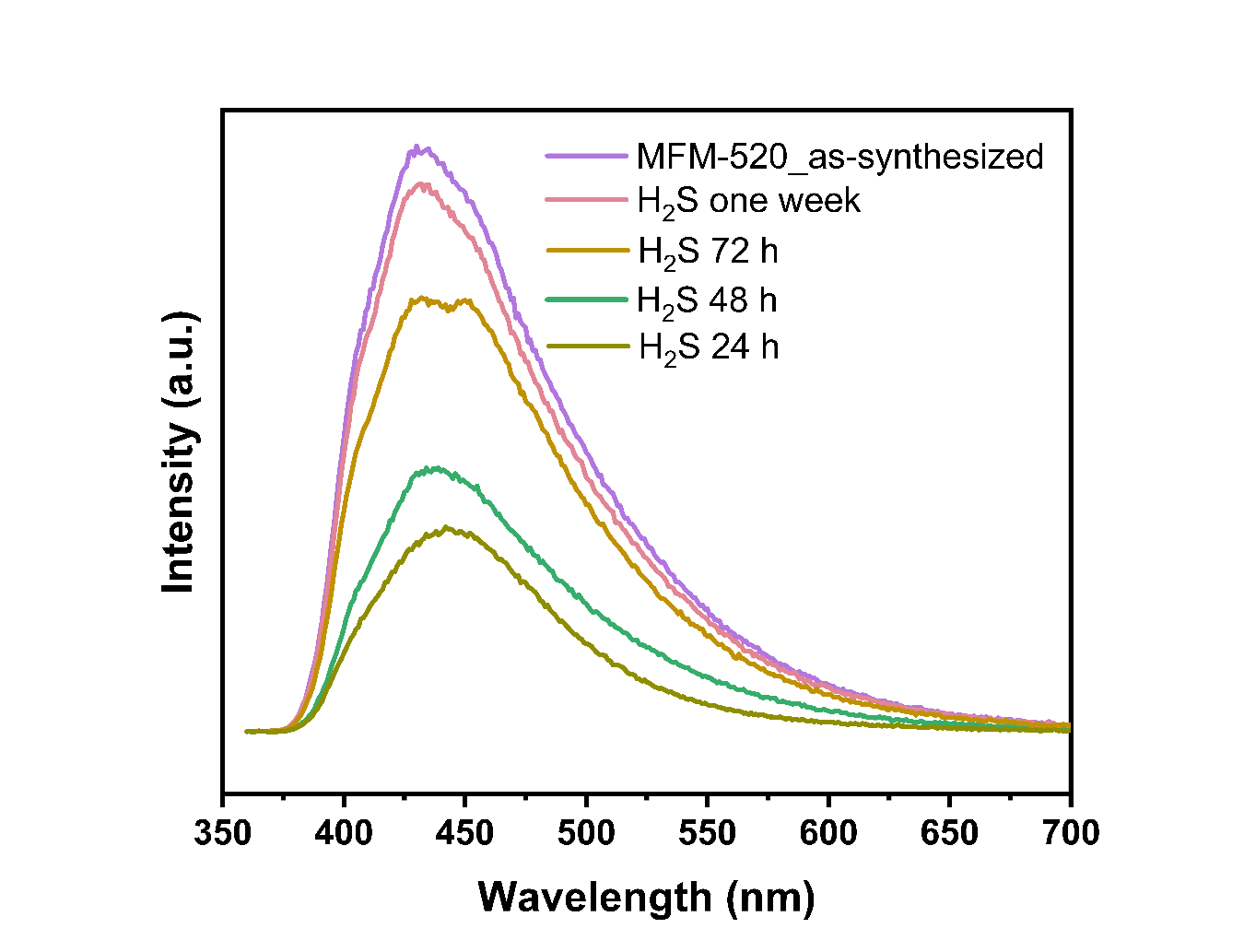


**Figure S8.** Solid-state PL spectra of the kinetic profile for H_2_S using MFM-520.


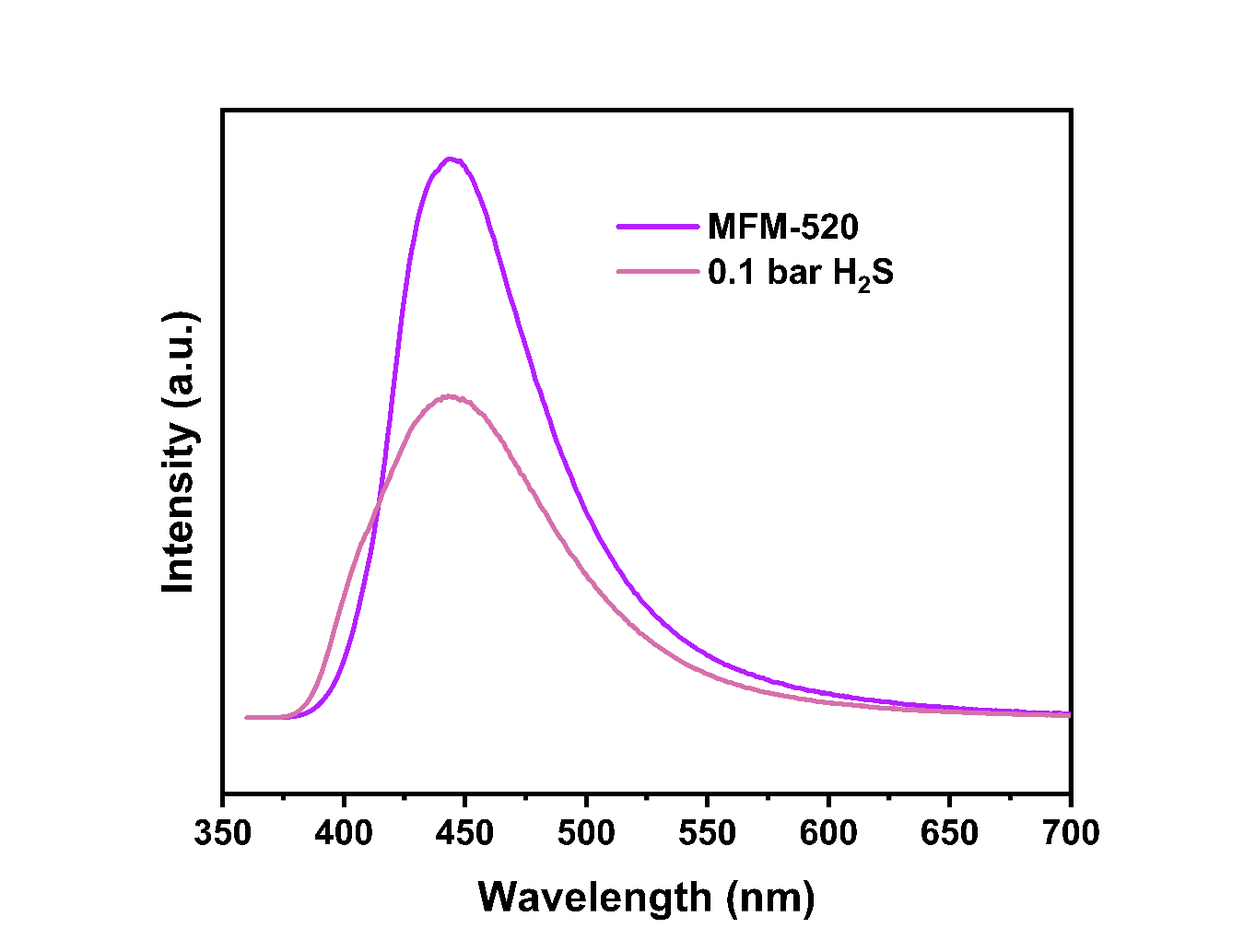


**Figure S9.** Solid-state PL spectra of MFM-520 saturated at 0.1 bar of H_2_S.


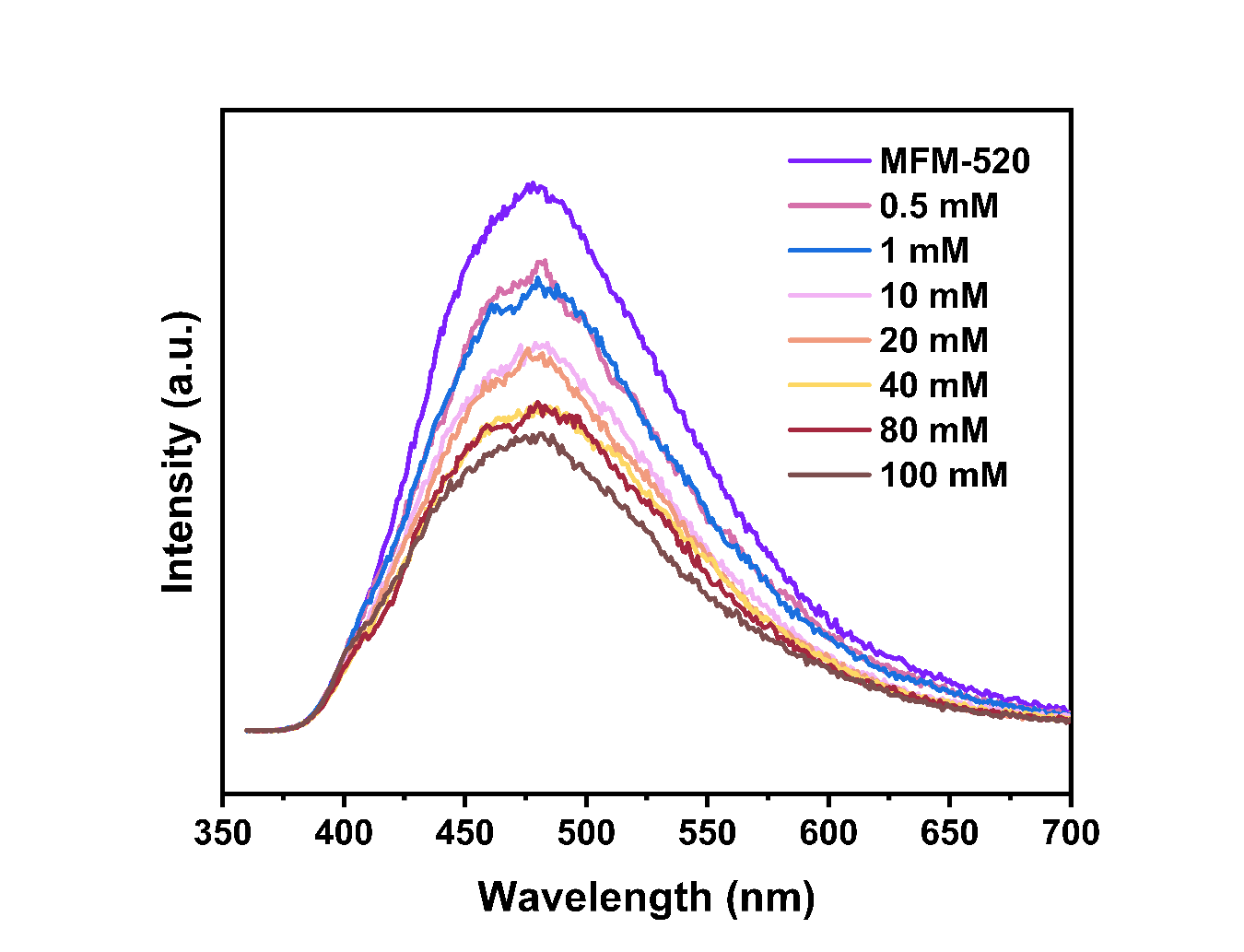


**Figure S10.** PL spectra of MFM-520 in H_2_S solution in THF at different concentrations.

**S4. Determination of the limit of detection (LOD)**

**
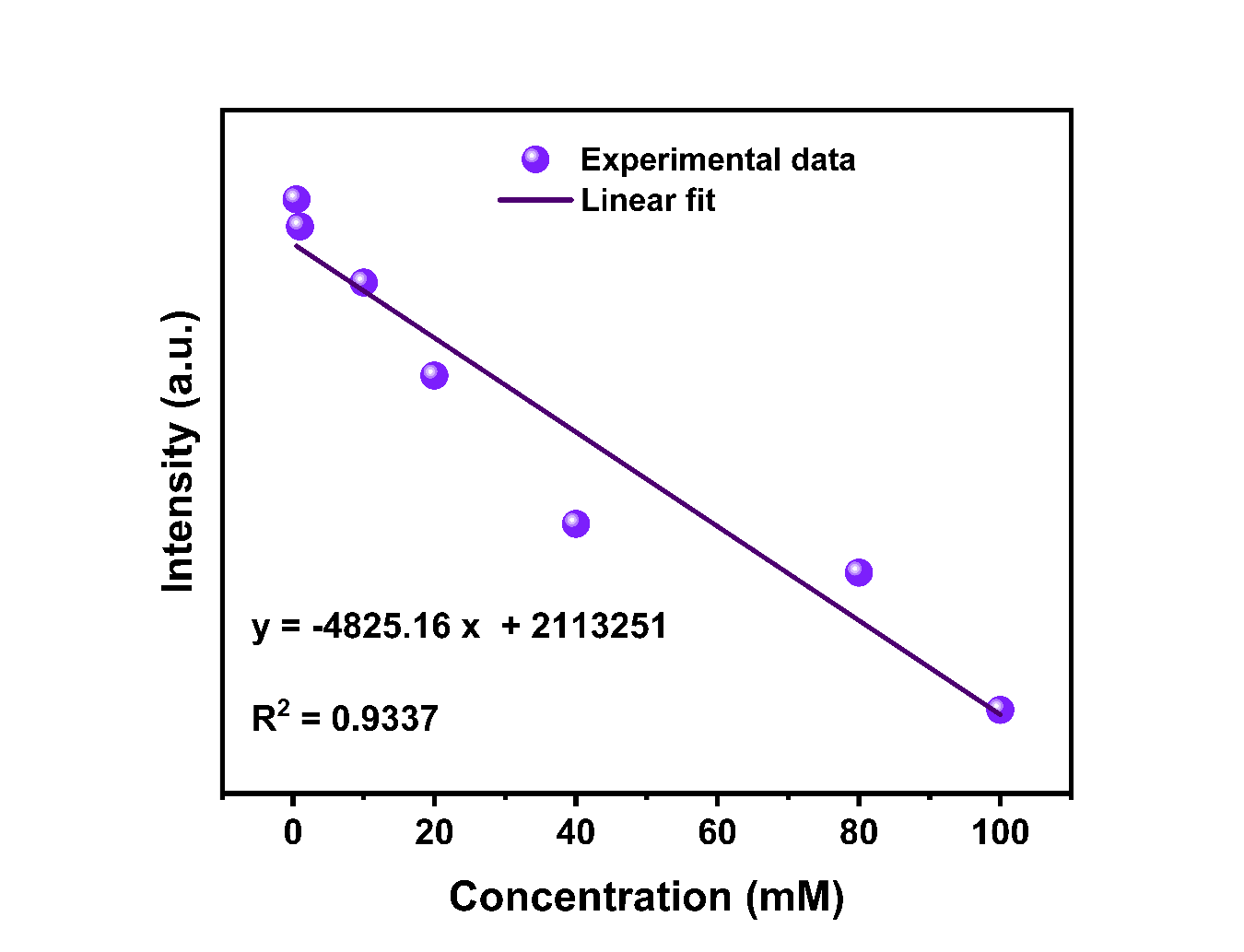
**

**Figure S11.** Calibration curve of H_2_S in THF solution for MFM-520.

The limit of detection (LOD) was calculated using the following equation:^[3]^

$$LOD =\frac{-3\sigma}{m} \boldsymbol{Eq.S}\boldsymbol{4}$$

Where σ is the standard deviation of blank readings and m is the slope of fluorescence intensity *vs.* H_2_S concentration plot.

The slope of the fluorescence intensity was determined by a linear fit of fluorescence intensity versus H_2_S concentration. Obtaining a line equation of:

y = −4825.16x + 263.190909

With a good correlation of R^2^ = 0.9337

To obtain the standard deviation (σ) of the pristine material reading, three aliquots of a 5 mg suspension of MFM-520 in 20 mL THF were taken and their emission spectra were measured The standard deviation (σ) was calculated using the intensities of those 3 blank readings.

Thus, the LOD was determined with the above data:

$Detection limit =\frac{-3\sigma}{m} = -\frac{-3\left( 289.270909 \right)}{-4825.16} = 0.18 mM$ $\boldsymbol{Eq.S}\boldsymbol{5}$

**S5. TRPL spectroscopy**

Fluorescence lifetimes were determined from the TPRL spectra. Data obtained from the decay spectra were globally fitted in Fluoracle software from Edinburgh Instruments, using a multi-exponential equation (Equation S6) to describe the fluorescence emission decay curve, through a reconvolution analysis with the instrument response function (IRF) measured under the same experimental conditions:^[4]^

$$R\left( t \right)=B{}_{1}e^{\left( \frac{-t}{\tau_{1}} \right)}+B{}_{2}e^{\left( \frac{-t}{\tau_{2}} \right)}+B{}_{3}e^{\left( \frac{t}{\tau_{3}} \right)} \boldsymbol{Eq.S}\boldsymbol{6}$$

where R(t) represents the fluorescence intensity as a function of time; B_1_, B_2_, B_3_ and B_4_ are the amplitudes of the respective decay components; and τ_1_, τ_2_ and τ_3_ are the lifetimes of the different components. Additionally, the χ^2^ value is reported.


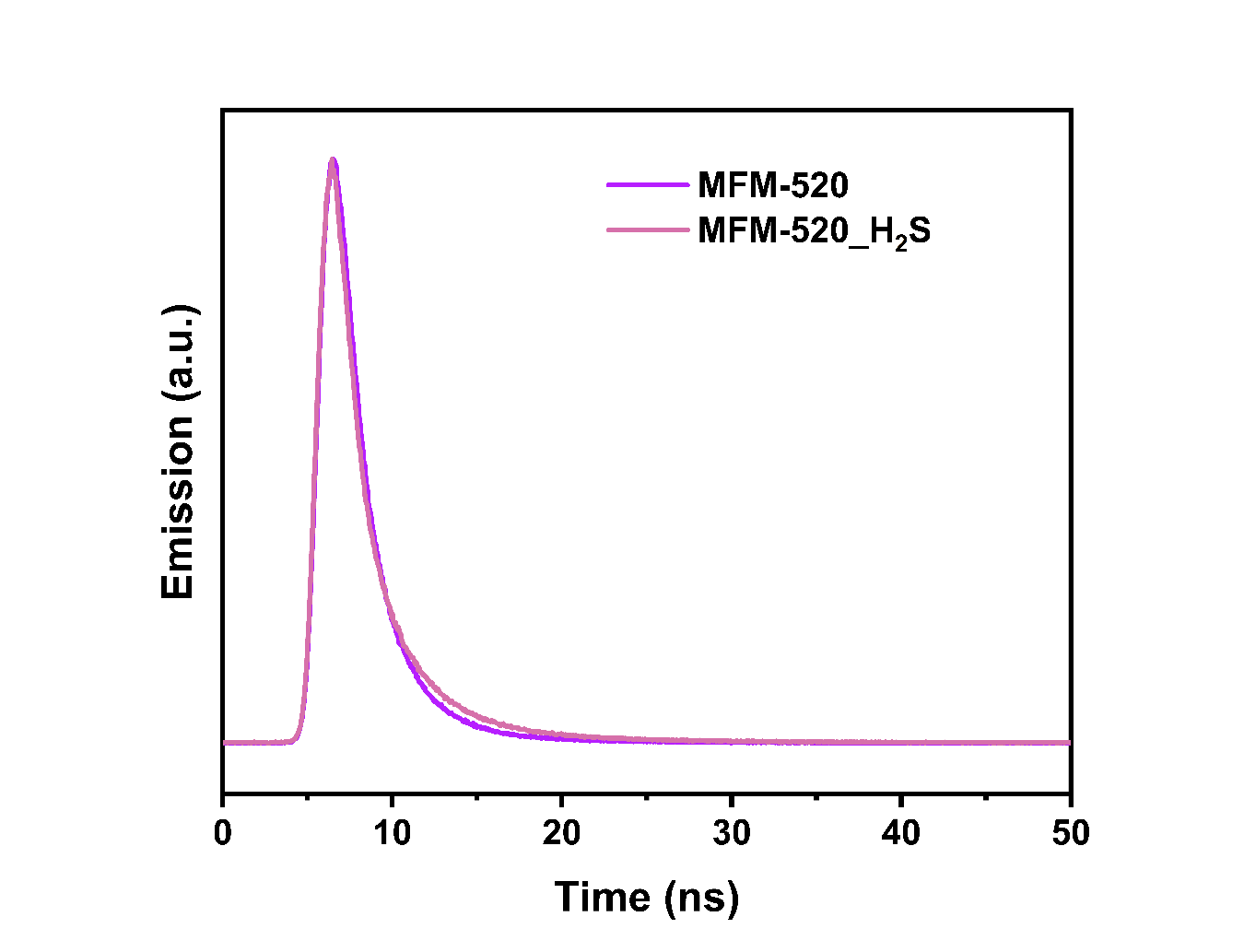


**Figure S12.** Time-resolved photoluminescence decay spectra of activated MFM-520 and H_2_S-saturated MFM-520 measured at 450 nm emission.

**Table S2.** Fluorescence lifetime in solid-state experiments

|  | Activated | Rel % | H_2_S-exposed | Rel % |
| --- | --- | --- | --- | --- |
| τ_1_ (ns) | 0.5447 | 21.50 | 0.5022 | 36.06 |
| τ_2_ (ns) | 2.6530 | 35.35 | 2.0379 | 33.67 |
| τ_3_ (ns) | 10.4819 | 43.15 | 9.7941 | 30.27 |
| Fluorescence lifetime (ns) | 5.57 | χ^2^ = 1.0936 | 3.83 | χ^2^ =1.0159 |


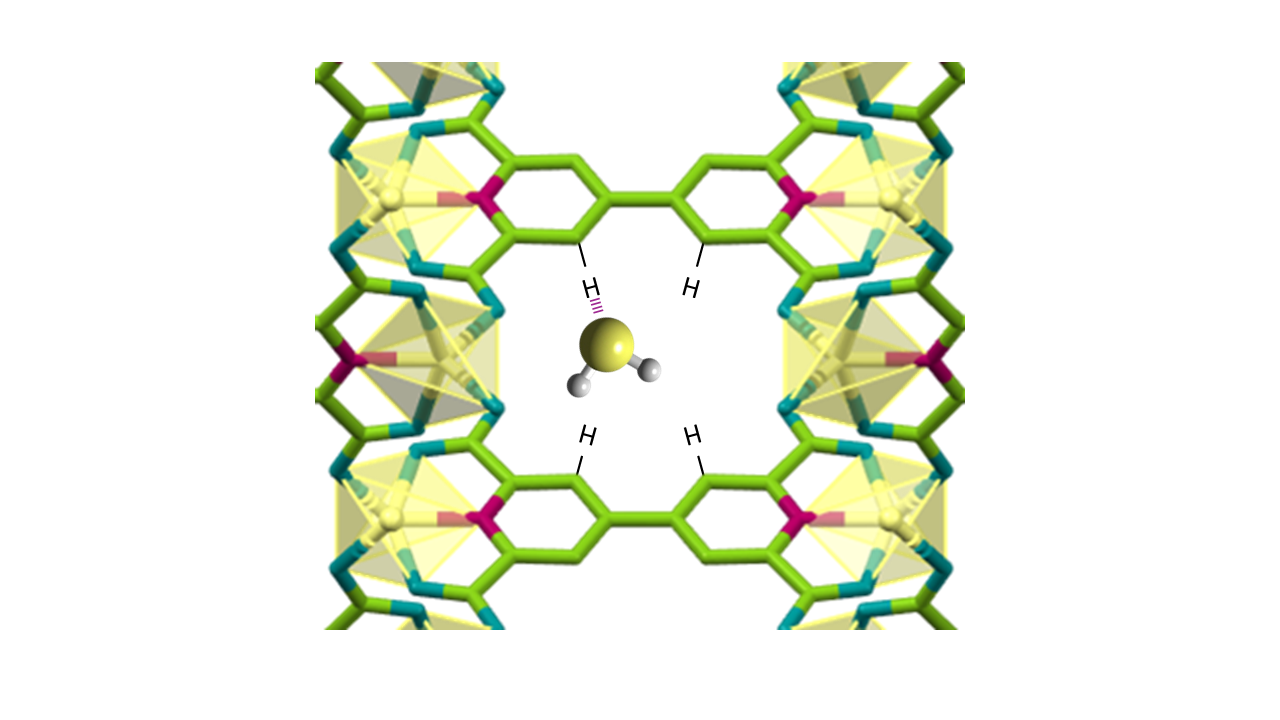


**Figure S13**. Schematic representation of H_2_S molecules confined within the ultramicroporous channels of MFM-520, illustrating proposed supramolecular interactions with the aromatic linker.

**S6- Quantum Yields (QY)**

The quantum yield (QY) measurements were carried out using an Edinburgh Instruments FS5 fluorimeter equipped with a continuous-wave 150 W ozone-free xenon arc lamp at room temperature. For solid-state measurements, the system was coupled with an SC-30 solid-state integrated sphere with a PTFA holder. These measurements were performed using the direct method. As an example, Figures S14-S16 present the spectrum of MFM-520 obtained directly from the Fluoracle software, illustrating the acquisition of the absolute quantum yield of the H_4_L acid ligand, the activated material, and the H_2_S-exposed condition.


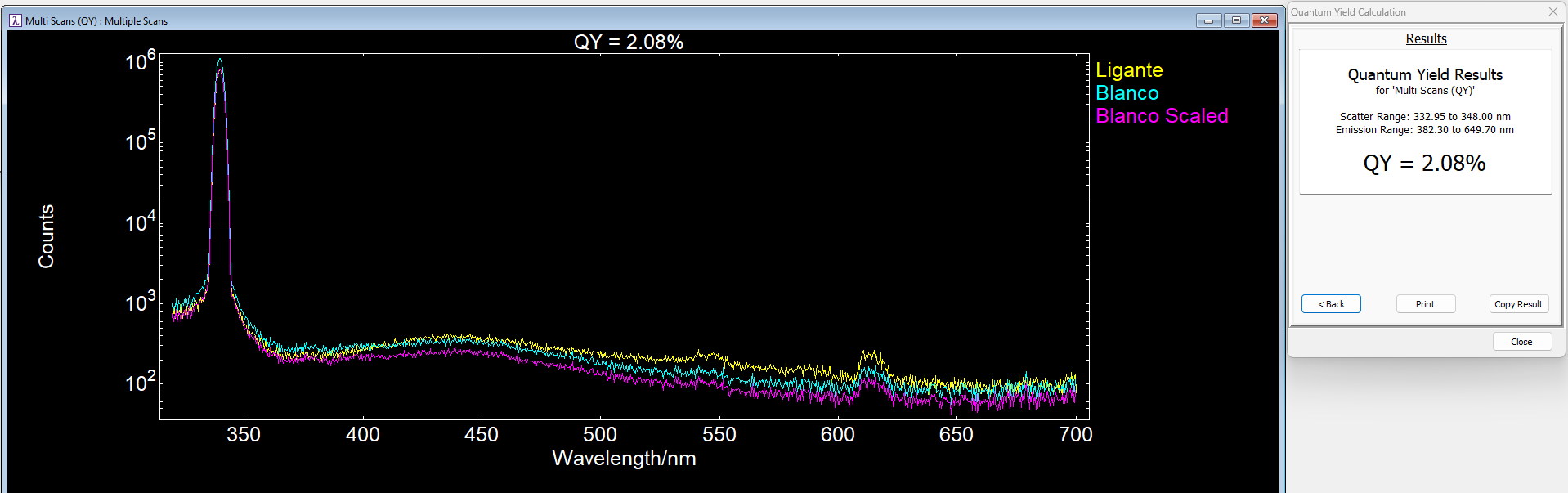


**Figure S14**. Absolute QY of the H_4_L acid ligand in the solid state, at λ_ex_ = 340 nm.


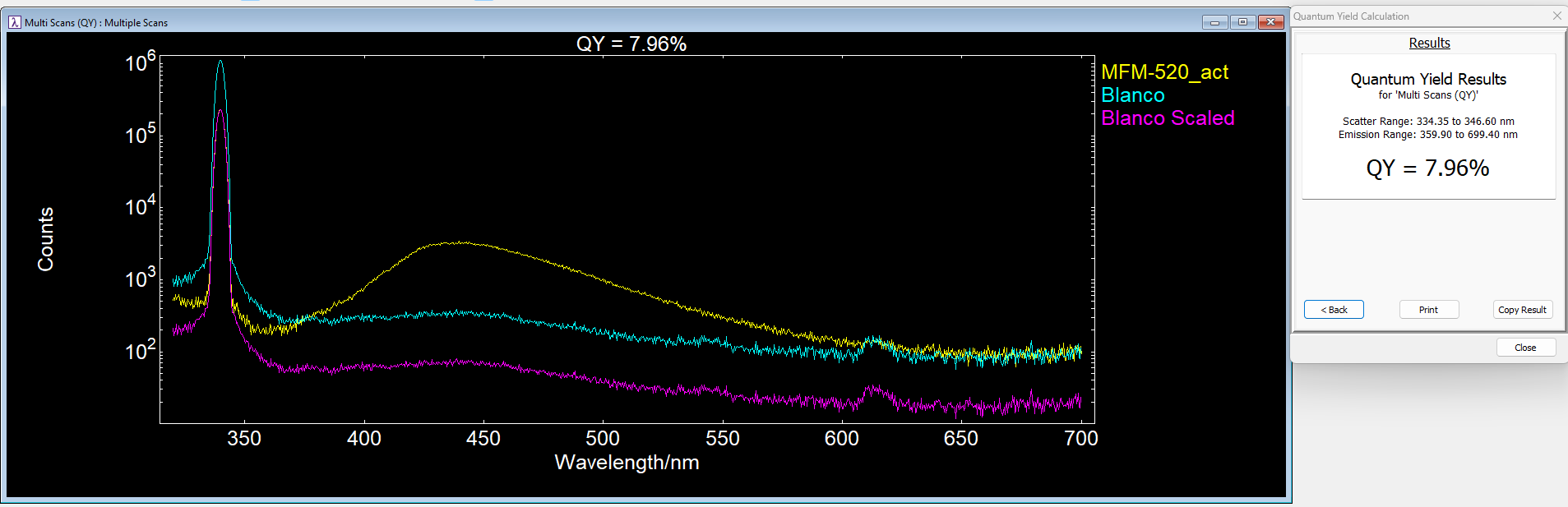


**Figure S15**. Absolute QY for the activated MFM-520 at λ_ex_ = 340 nm.


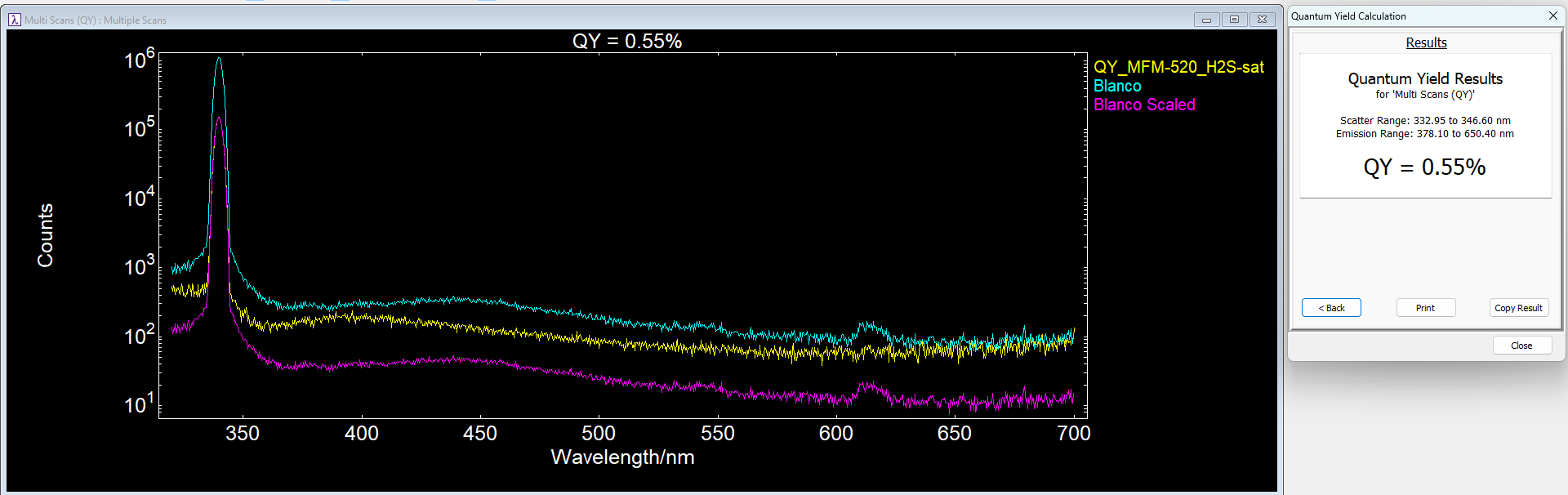


**Figure S16**. Absolute QY for the H_2_S saturated MFM-520 at λ_ex_ = 340 nm.

**Table S3.** Comparison of representative MOF-based H_2_S sensors.

| **Material** | **Medium** | **Sensing type** | **LOD** | **Response time** | **Reversi-bility** | **Stability** | **Ref.** |
| --- | --- | --- | --- | --- | --- | --- | --- |
| Zr-pydc-Eu | Aqueous | Turn-on | μM range | Fast | Yes | High | [5] |
| DUT-52-N_3_ | Aqueous | Turn-on | 0.50 μM | 2 min | Yes | High | [6] |
| MIL-125-NH_2_-  bpydc | Aqueous  /biological | Turn-on | 0.33 nM | Fast | Yes | Improved (PSM) | [7] |
| Zr(TBAPy)_5_  (TCPP) | Aqueous  /gas (paper) | Turn-on | ppb level | 10 s | Yes | High | [8] |
| Al-MIL-53-NO_2_  MMM | Gas  (membrane) | Turn-on | nM range | Fast | Yes | Moderate | [9] |
| Zn-MV-BTEC  MOF | Gas  (natural gas) | Turn-off | 0.95 ppm | Moderate | Not fully discussed | Moderate | [10] |
| MTV-CAU-10 | Aqueous | Turn-on | 110 nM | Ultra-fast | Not | High | [11] |
| Zn-bdc | Aqueous | Turn-off | 10.7 μM | Low | Not | High | [12] |
| MIL-100(Fe) | Solid state | Turn-on | 0.43 ppm | 278 s | Not | Moderate | [13] |
| MOF-919 |  |  | 0.31 ppm | 144 s | Yes | High |  |
| HKUST-1 |  |  | 0.47 ppm | 28 s | Not | Moderate |  |
| **MFM-520 (this work)** | **Gas**  **(solid state/aqueous)** | **Turn-off** | **6.13 ppm** | **Minutes** | **Yes** | **High** | **This work** |

**S7. References**

[1] E. Sánchez-González, P. G. M. Mileo, M. Sagastuy-Breña, J. R. Álvarez, J. E. Reynolds, A. Villarreal, A. Gutiérrez-Alejandre, J. Ramírez, J. Balmaseda, E. González-Zamora, G. Maurin, S. M. Humphrey, I. A. Ibarra, *J. Mater. Chem. A* **2018**, *6*, 16900–16909.

[2] J. A. Zárate, E. Sánchez-González, T. Jurado-Vázquez, A. Gutiérrez-Alejandre, E. González-Zamora, I. Castillo, G. Maurin, I. A. Ibarra, *Chem. Commun.* **2019**, *55*, 3049–3052.

[3] A. Sharma, D. Kim, J. H. Park, S. Rakshit, J. Seong, G. H. Jeong, O. H. Kwon, M. S. Lah, *Commun. Chem.* **2019**, *2*, 1.

[4] U. Noomnarm, R. M. Clegg, *Photosynth. Res.* **2009**, *101*, 181.

[5] Y. Shu, J. Hao, D. Niu, Y. Li, *J. Mater. Chem. C*, **2020**, 8, 8635-8642.

[6] C. Gogoi, A. Kumar, M. SK, S. Biswas, *Microporous and Mesoporous Materials*, **2021**, 311, 110725.

[7] Z. Weng, Z. Xie, X. Wu, B. Qiu, J. Chen, W. Sun, Z. Lin, *Small Methods,* **2025**, 9, 2500277.

[8] L. Guo, M. Wang, D. Cao, *Small,* **2018**, 14, 1703822.

[9] X. Zhang, Q. Zhang, D. Yue, J. Zhang, J. Wang, B. Li, Y. Yang, Y. Cui, G. Qian, *Small,* **2018**, 14, 1801563.

[10] R. Song, L. Hou, Y. Wang, Y. Li, X. Wang, Y. Zang, Y. Zang, X. Wang, S. Yan, *Anal. Methods* **2017**, *9*, 3914.

[11] S. Nandi, H. Reinsch, S. Biswas, *Microporous and Mesoporous Material*s **2020**, *293*, 109790

[12] M. D. Dawn, K. Nath, S. Saha, P. K. Roy, M. Mandal, K. Biradha, *Mater. Adv.* **2023**, *4*, 5730–5739.

[13] K.-H. Luo, H.-W. Tai, D. K. D. Chittibabu, K. S. Santiago, Z.-K. Ni, S.-H. Yang, H.-T. Chen, C.-H. Lin, J.-M. Yeh, *Sens. Actuators, B* **2025**, *443*, 138200.
